# Polyanion Chemistry Engineers Ternary RNA Nanoparticle Structure/Function from the Inside-Out

**DOI:** 10.1021/acsnano.5c19683

**Published:** 2026-01-27

**Authors:** Lijun Hu, David J. Peeler, Tianyi Jin, James J. Doutch, Baihao Shao, Jonathan Yeow, Li Ma, Hanna M. G. Barriga, Jiaqing Tang, Xuan Cao, Chenchen Liu, Christopher L. Grigsby, Alfredo Alexander-Katz, Robin J. Shattock, Molly M. Stevens

**Affiliations:** † Kavli Institute for Nanoscience Discovery, Department of Physiology, Anatomy and Genetics, Department of Engineering Science, University of Oxford, Oxford OX1 3QU, United Kingdom; ‡ Department of Materials, Department of Bioengineering, Institute of Biomedical Engineering, 152938 Imperial College London, London SW7 2AZ, United Kingdom; § Department of Infectious Disease, Faculty of Medicine, Imperial College London, London SW7 2AZ, United Kingdom; ∥ Department of Chemical Engineering, 2167 Massachusetts Institute of Technology, Cambridge, Massachusetts 02139, United States; ⊥ ISIS Neutron and Muon Source, Harwell Campus, Oxford OX1 10QX, United Kingdom; # Department of Medical Biochemistry and Biophysics, 27106 Karolinska Institute, Solna, Stockholm 171 77, Sweden; ∇ Department of Earth Sciences, University of Oxford, Oxford OX1 3AN, United Kingdom; ○ Department of Earth Sciences, University of Cambridge, Cambridge CB2 3EQ, United Kingdom; ◆ Department of Materials Science and Engineering, Massachusetts Institute of Technology, Cambridge, Massachusetts 02139, United States

**Keywords:** self-amplifying RNA, PET-RAFT, SANS, high throughput, structure/function, molecular dynamics

## Abstract

Formulating cationic polyplexes (PP) with polyanions as ternary polyelectrolyte nanoparticles (TNP) offers a polymeric alternative to lipid nanoparticles (LNP) for targetable nucleic acid delivery. Although TNP *in vivo* transport is credited to their anionic surface charge, the relationships between polyanion chemistry and TNP structural stability, protein binding, and transfection are poorly understood compared to lipid-based systems. We hypothesized that carefully engineered hydrophobic polyanions could simultaneously endow TNPs with negative surface charge and enhanced extracellular stability critical to the future development of actively targeted formulations. We synthesized chemically diverse PEGylated polyanions to coat self-amplifying RNA (saRNA) PP, systematically studying how PEG architecture and polyanion chemistry modulate TNP structure and function. In both high-throughput stability assays and Small Angle Neutron Scattering structural studies, we found that PEG_5k_-*bl*-polyanion_5k_ yields remarkably small particles with a pH-responsive core–shell structure. We identify a lead formulation (TNP5) with moderate hydrophobicity and charge density that balances extracellular stability and intracellular unpackaging for transfection. In agreement with spectroscopic characterization and *in vitro* cell studies, Molecular Dynamics simulations support the hypothesis that polyanions dictate TNP function from the inside-out by excluding water from the RNA core and by exposing functional groups that modulate protein binding. Our work correlates high throughput assays and detailed neutron scattering analysis to uncover mesoscale structural differences between two- and three-component polyelectrolyte delivery systems. These screening methods and the critical balances between polymer properties they uncover establish a framework for high throughput engineering of pH-responsive nanoparticle structure/function to navigate biological barriers to RNA delivery.

## Graphical abstract

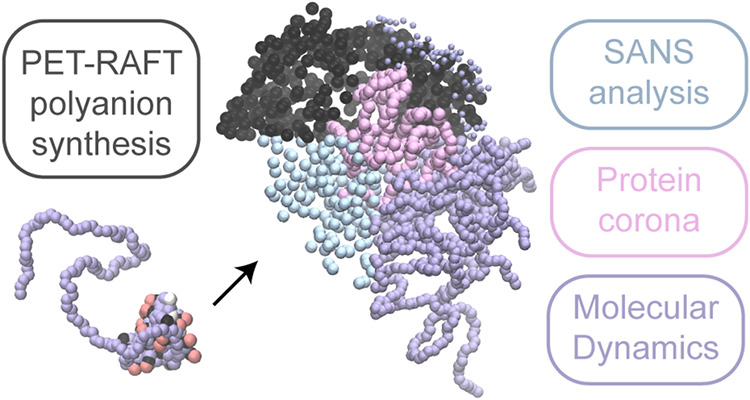

## Introduction

There is a significant need to understand how the chemistry and nanoscale structure of mRNA carriers alter interactions with physiological barriers that dictate transfection outcomes. For example, while inhalable lipoplex,^1^ lipid nanoparticle (LNP)^2–4^ and polyplex (PP)^5,6^ mRNA vaccine formulations have been identified, rational material design to enhance local antigen presenting cell (APC) transfection is limited by our understanding of structure/function relationships in mucosal tissues. Tailoring nanomaterial chemistry to enable transfection in particular cell types requires understanding dynamic physicochemical responses to both extracellular (*e.g.*, protein binding) and intracellular (*e.g.*, pH) interactions.^7^ Although many studies show that diffusive transport is most efficient for small nanoparticles with hydrophobically stabilized cores and densely sterically stabilized shells,^8^ how steric hindrance (*e.g.*, PEGylation strategies) and surface charge alter the structure, biological interactions, and transfection efficiency of mRNA delivery vehicles remain incompletely understood.^9^

The dynamics and resilience of LNP and PP steric stabilization depend on the molecular weight, branching architecture, and conjugation chemistry of the steric stabilizer used. For example, both the molecular weight of oligo­(ethylene glycol)^10^ and carboxybetaine^11^ brush polymers and the length of their conjugated diacyl chains were shown to impact protein-mediated displacement from the LNP surface. In contrast to the biomimetic lipoprotein-like physicochemical properties of LNPs, fully synthetic polyelectrolyte-RNA formulations offer a highly cross-linked structure amenable to both direct^5,12^ and supramolecular^13–18^ polycation PEGylation strategies. Although widely reported to increase nuclease resistance *in vitro*, PEG may also impart flexibility and hydration to the amorphous, cationic PP core, increasing the ease with which negatively charged biomolecules can unpackage nucleic acid cargo.^19^ And despite near-neutral zeta potential measurements, PEGylated polycations inherently position amines at the PP surface that may limit active targeting strategies by promoting nonspecific proteoglycan uptake mechanisms.^20^ These contradictions demand a better understanding of how PEGylated polyelectrolytes influence cargo packaging, colloidal stability, and cellular uptake of polymeric RNA formulations.

Electrostatically coating cationic nanoparticles with anionic polymers offers an alternative supramolecular approach to improve particle stability by simultaneously reprogramming surface charge and increasing intraparticle cross-linking.^21^ Ternary polyelectrolyte complex nanoparticles (TNP) maintain the ethanol-free, lyophilization-compatible advantages of PP formulations while presenting an electronegative surface charge to enable ligand-mediated transfection *in vivo*.^22–26^ Polyanions with polyacrylate,^21^ polyacrylamide,^27,28^ polypeptide (*e.g.*, poly­[glutamic acid] ^29,30^ ), and polysaccharide (*e.g.*, hyaluronic acid^31^) backbones have been formulated for TNP delivery of pDNA, siRNA, mRNA, and self-amplifying mRNA (saRNA). PEGylated polyanions^28,31^ have been shown to promote small TNP size (<100 nm diameter), negative zeta potential, and protection from nuclease attack, but systematic variations of polyanion chemistry remain essentially unexplored compared to polycation^32,33^ and lipid^34,35^ combinatorial chemistry strategies. Moreover, despite an interest in blending polyelectrolytes to alter both TNP and LNP structure/function,^32,33,36,37^ such correlation is limited by a lack of *in situ* biophysical characterization of mesoscale structural features that are known to underlie the function of PP and LNP systems.^38–43^

In this work, we used high throughput polymer chemistry, Small Angle Neutron Scattering (SANS) structural analysis, and molecular modeling to investigate how PEG architecture and polyanion chemistry (charge density, hydrophobicity, and molecular weight) shape saRNA TNP structure/function dynamics. Building on our past work, we used saRNA PP formed with the reducible polycation poly­(cystamine bis­(acrylamide)-*co*-4-amino-1-butanol) (pABOL) as the base of our formulations to leverage its transfection potency and moderate hydrophobicity.^44^ We established a well plate-based workflow for high throughput synthesis of coating polymers (*via* photoinduced electron/energy transfer reversible addition–fragmentation chain transfer, PET-RAFT^45–47^ ), aqueous nanoparticle library formulation, and screening of size stability (*via* Dynamic Light Scattering, DLS), saRNA packaging (*via* Förster Resonance Energy Transfer, FRET), and cellular uptake/transfection. We targeted densely PEGylated particles ∼50–75 nm in diameter with neutral or slightly negative surface charge to enable efficient transport in physiological environments. Screening revealed that PEG_5k_-*bl*-polyanion_5k_ architectures yield remarkably small (∼50 nm) anionic TNP with tunable properties, with polyanion hydrophobicity offsetting instability induced by polycation deprotonation. SANS investigations and Molecular Dynamics (MD) simulations elucidated previously unexplored mechanisms of polyanion-mediated structure–function changes in TNP systems, most notably that polyanion hydrophobicity governs tight core packaging while creating solvent-accessible apolar surfaces that influence protein binding. These structural characteristics directly influenced cellular interactions, where more hydrophobic particles showed reduced uptake in protein-free conditions but exhibited serum-enhanced uptake and serum-compromised RNA release. By integrating high throughput, biophysical, computational, and intracellular techniques, we reveal structure/function principles to engineer polyelectrolyte nanoparticles that dynamically navigate barriers to RNA delivery.

## Results and Discussion

### Diblock PEG_5k_-Polyanion_5k_ Reprogram PP into Colloidally Stable Charge-Neutralized TNP

The high negative charge density (∼320 *m*/*z* ^–^) of RNA’s phosphate backbone facilitates electrostatic complexation with the tertiary amine backbone of pABOL (∼386 *m*/*z* ^+^) and thus polyplex (PP) condensation. Formulation with excess polycation (pABOL:saRNA 45:1 w/w) drives efficient transfection^44^ but also electrostatic binding to ubiquitous glycosaminoglycans that limit strategies to increase cell-type specificity.^48^ We hypothesized that polyanions with optimized PEG architecture, charge density, and hydrophobicity could enable ligand-targeted delivery by neutralizing PP surface charge without displacing saRNA.^49^ We thus synthesized a library of polyanions with systematically varied chemistries using high-throughput PET-RAFT polymerization (Scheme S1). These polymers (general structure shown in Figure 1) were designed with two functional segments: (i) a hydrophilic linear or brush PEG outer shell-forming segment and (ii) a second PP surface-binding segment composed of copolymerized 2-carboxyethyl acrylate (CEA, anionic, cationic PP binding), butyl acrylate (BA, hydrophobic, stabilizing), and 2-hydroxyethyl acrylate (HEA, hydrophilic, spacing). The library consisted of four polyanion series with varying PEG lengths, architectures, and block compositions. The first three series feature variations in PEG lengths and architectures of linear PEG_5k_-*bl*-polyanion (P1–P9, Table 1), linear PEG_2k_-*bl*-polyanion (P10–P18, Table S1), and brush pOEGA_5k_-*st*-polyanion (P19–P27, Table S1). Initial attempts to synthesize pOEGA_5k_-*bl*-polyanion block copolymers resulted in poor solubility and formation of larger ternary particles, leading us to pursue the statistical copolymer architecture for the brush PEG series. Within each series, the second segment is fixed at a molecular weight of 5 kDa, with systematic variation in composition targeting CEA incorporations of 25, 50, or 75% and BA incorporations ranging from 0 to 75%. The fourth series features a constant linear PEG_5k_ conjugated to second blocks with molecular weights ranging from 10 to 40 kDa. This molecular weight series consists of eight polymers with two distinct compositions, P28–P31 incorporating CEA and HEA at a 50:50 molar ratio, and P32–P35 incorporating CEA, HEA, and BA at a 50:25:25 molar ratio (Table S1).

**Figure 1:**
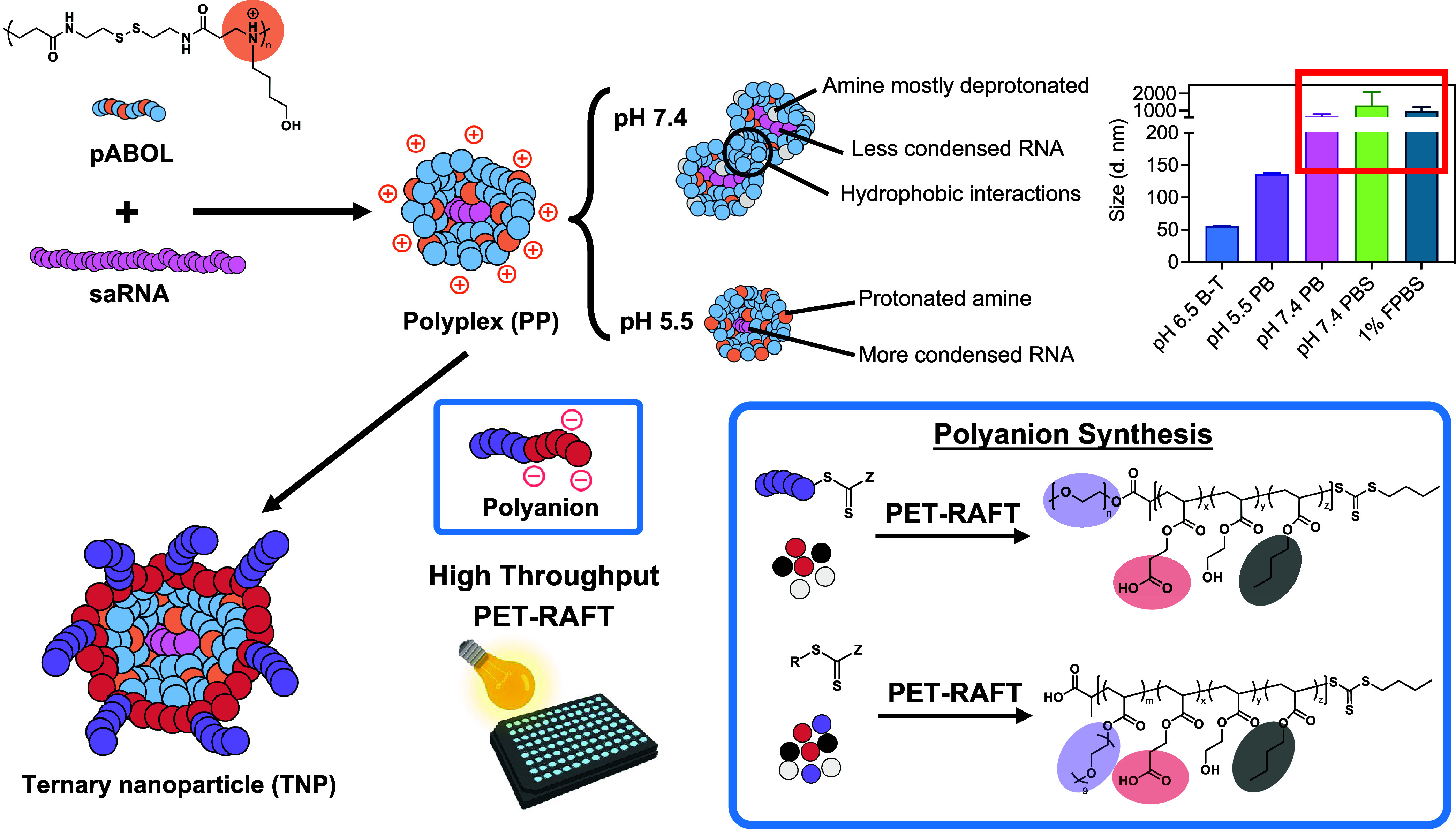
Design of a combinatorial saRNA ternary nanoparticle (TNP) library. Schematic showing TNP formation through electrostatic coating of pABOL polyplexes (PP) with polyanions of diverse chemical properties. Light bulb and well-plate adapted from BioRender.com. The graph shows DLS measurements of the intensity-weighted mean hydrodynamic diameter of PP in different buffer conditions, demonstrating significant aggregation and instability in physiological pH and protein-rich environments. Data shown as mean ± SD, *N* = 3.

**Table 1:**
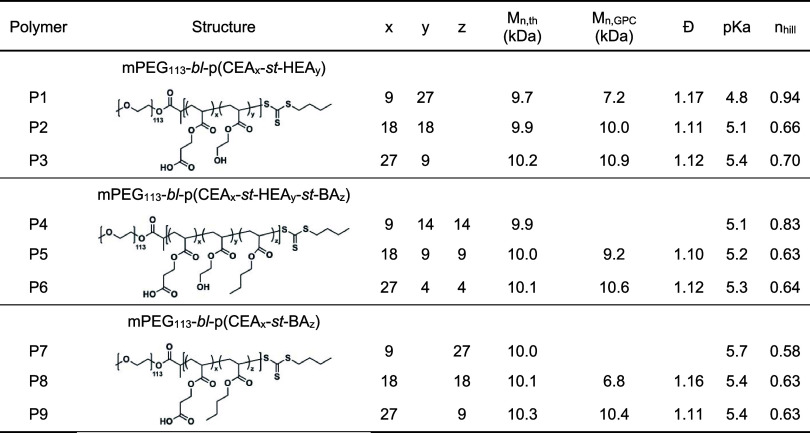
PEG_5k_-*bl*-Polyanion_5k_ Library Characterization Using NMR, GPC, and Titration

Synthesis of polyanions *via* PET-RAFT provided good control of polyanion composition (confirmed by ^1^H NMR; Figures S1–S4, Tables 1, and S1) and molecular weight dispersity (Đ, confirmed by aqueous GPC; Figure S5). Titration studies revealed that p*K*_a_ values for P1–P9 ranged from 4.8 to 5.7, demonstrating significant compositional influence (Figure S6A, and Table 1). Increasing anionic CEA and hydrophobic BA content elevated p*K*_a_ values (*e.g.*, p*K*_a_ increased from 4.8 for P1 to 5.4 for P3, and 5.7 for P7). Notably, these p*K*_a_ values are higher than that of RNA (∼2) and lower than that of pABOL (7.1, Figure S7A). Polymer cooperativity analysis using Hill plots (Figures S6B, and S7B) revealed consistently low *n*_Hill_ values (*n*_Hill_ < 1), indicating noncooperative behavior, even for polyanions with higher molecular weights (10–40 kDa, P31–P35) that enhance this phenomenon for polycations.^50,51^ Thus, both pABOL and coating polymers will contribute to the buffering capacity of TNPs as they undergo pH-responsive protonation/deprotonation in physiological environments. Knowing that copolymerization of ionizable and hydrophobic monomers can endow pH-responsive particle stability and membrane disruption associated with efficient endosomal escape,^52,53^ we assessed pH-dependent lysis of human red blood cells (Figure S8). Only the most hydrophobic coating polymer P7 (25% CEA, 75% BA) showed undesirable hemolytic activity at extracellular pH 7.4 (EC_50_ ≈ 64 μM). And while hydrophobicity correlated with hemolytic activity at pH 5.5 as expected, only P8 (50% CEA, 50% BA) exhibited potency (EC_50_ = 12.5 μM) on an order of magnitude expected to significantly enhance endosomal escape.^52,53^ These results confirm the biocompatibility of carboxyethyl/butyl acrylate copolymers but also motivate future work with hydrophobic acidic monomers (*e.g.*, propylacrylic acid) to enhance endosomal escape functions.

We next investigated how PEG-polyanion chemistry (PEG architecture, charge density, hydrophobicity, and molecular weight) influences pABOL/saRNA TNP size and surface charge (Figure 2A,B). Base PP were prepared at a mass ratio of 45:1 pABOL:saRNA (molar charge ratio [N/P] = 37) optimized in previous work.^44^ By lowering buffer pH (20 mM Bis-Tris pH 6.5 vs 20 mM HEPES pH 7.4) to ensure pABOL protonation, we achieved PP with a small hydrodynamic size of 55.9 ± 0.4 nm and a zeta potential of 27.4 ± 2.2 mV with simple vortex mixing. The amount of polyanion added (molar charge ratio [C/N]) was optimized to neutralize surface charge while maintaining small particle size (Figure 2B). For linear PEG_5k_-*bl*-polyanion_5k_ coatings (TNP1 to TNP9), C/N = 1 was sufficient, whereas PEG_2k_-*bl*-polyanion_5k_ coatings (*e.g.*, P11, P14, P17, which have the same polyanion composition as P2, P5, P8, respectively) required C/N = 1.5 to reach the same TNP size. Brush pOEGA_5k_-*st*-polyanion_5k_ coatings (*e.g.*, P20, P23, P26) necessitated substantially higher C/N ratios (C/N = 3) to maintain ∼100 nm size, perhaps due to limited accessibility imparted by their PEG architecture. Polyanions with longer anion blocks (10–40 kDa, P31–P35) produced larger particles with incomplete charge neutralization at C/N = 1. This behavior may be attributed to two factors: the reduced flexibility of longer anionic blocks in aqueous solution, which limits their ability to rearrange and bind efficiently interfering with binding to the PP core, and the lower PEG:anion ratio of these polymers. These results demonstrate that diblock architectures with equal molecular weights of each block (PEG_5k_-*bl*-polyanion_5k_) facilitate efficient formulation of small and charge-neutralized TNPs; to our knowledge, these are the first reported saRNA TNPs with negative zeta potential.^17,30^ They also establish PEG-polyanion design rules: PEG length is critical for steric stabilization and sufficient to accommodate a variety of anionic segments, whose length must be minimized to allow for neutralization and sufficient PEG density. Although these data and past work with siRNA^54^ suggest that higher molecular weight PEGs or shorter polyanions could also stabilize saRNA TNPs, we chose to focus on understanding how polyanion chemistry influences RNA packaging and the biological interfacing of TNPs stabilized with the symmetric PEG_5k_-*bl*-polyanion_5k_ family identified.

**Figure 2:**
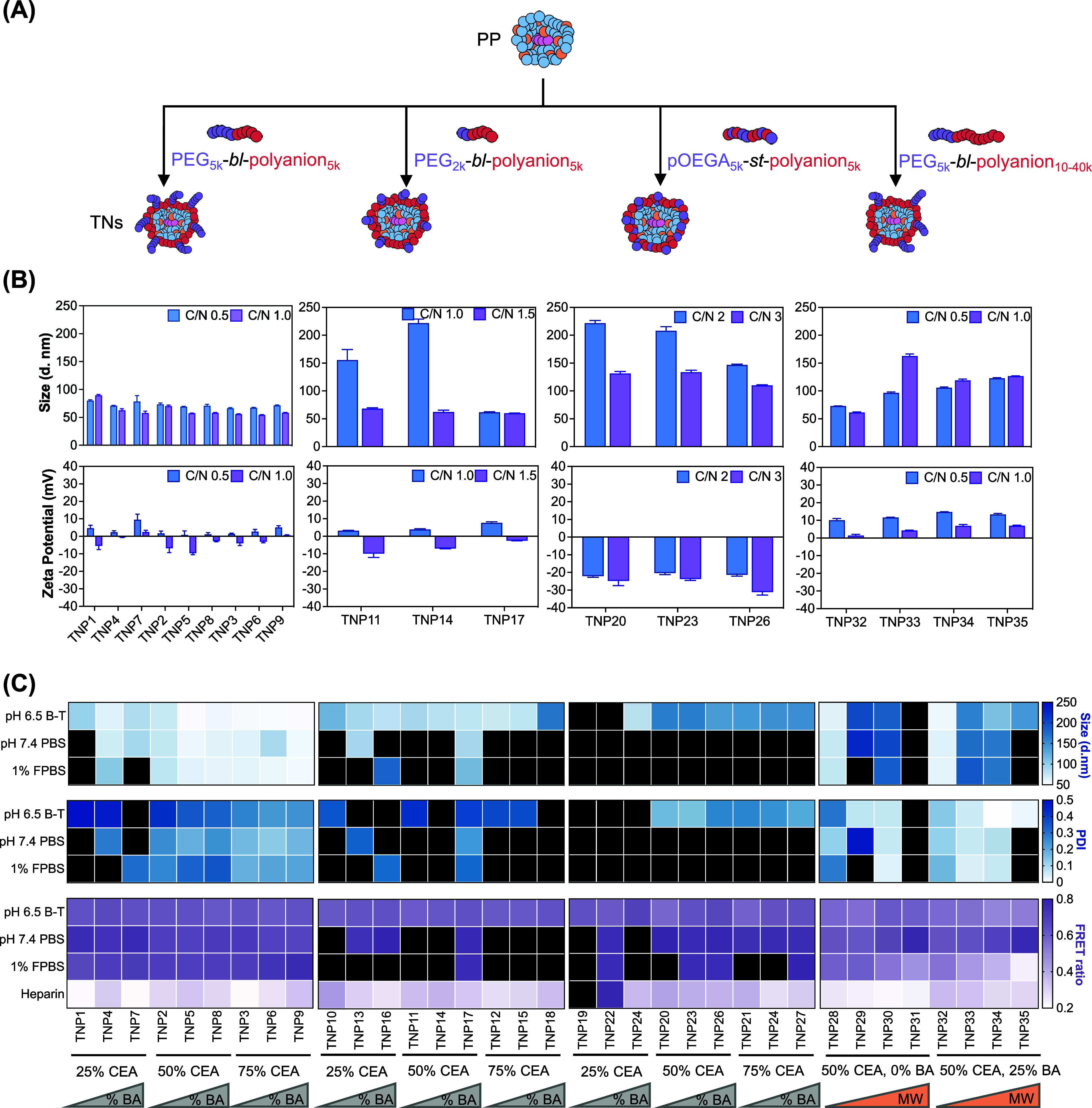
Polyanion PEG architecture, charge density, hydrophobicity, and molecular weight dictate TNP size, surface charge, and stability. (A) Schematic overview of PEG-polyanion architectures used in TNP libraries. (B) Optimization of TNP hydrodynamic diameter and zeta potential by varying C/N ratio for each polymer. Data shown as mean ± SD, *N* = 3. (C) Colloidal size (intensity-weighted mean hydrodynamic diameter) and RNA packaging stability of TNP formulations at optimized C/N ratios. Heatmap in black indicates exceeding the upper range. Data shown as mean of three replicates, *N* = 3.

We then assessed how polyanion chemistry altered TNP colloidal stability in the face of physiological “challenges” represented by salt, protein, and heparin. A library of 35 TNPs was formulated with different polyanions at their optimal C/N ratios, followed by incubation in formulation buffer (Bis-Tris, B-T), PBS with or without 1% fetal bovine serum (1% FPBS) at 37 °C for 4 h where particle size distribution and saRNA packaging were monitored using DLS and FRET, respectively (Figure 2C). As expected, PP rapidly aggregated in salt and protein-rich conditions (Figure 1). Among the four TNP series examined, those coated with linear PEG_5k_-*bl*-polyanion_5k_ (P1–P9) exhibited superior particle size stability, while TNP coated with PEG_2k_-*bl*-polyanion_5k_ (P10–P18) or brush pOEGA_5k_-*st*-polyanion_5k_ (P19–P27) showed significant aggregation similar to uncoated PP. Increasing anionic block length (P28–P31, P32–P35) progressively diminished stability, with 40 kDa block lengths (P31 and P35) forming aggregation in both PBS and 1% FPBS. These results echo that of initial formulation characterization above: matching the length of the hydrophilic linear PEG segment with the anionic segment provides optimal steric stability. Within the PEG_5k_-*bl*-polyanion_5k_ series, we discovered that particle stability depends on a complex interplay of factors beyond PEG content alone, with the anion block composition playing a crucial role through a delicate balance of properties: TNPs formulated with the lowest charge density polyanion (P1; ∼555 *m*/*z* ^–^) formed aggregates in both PBS and FPBS conditions despite conveying high PEG density; at the other extreme, the most hydrophobic TNP (P7) also showed instability, forming aggregates in FPBS. Thus, optimal TNP stability also requires a balanced composition of charge density and hydrophobicity in the polyanion block.

The extents to which formulations prevent RNA degradation by RNase or RNA release by heparin are commonly used to benchmark functional stability.^50,55^ Using gel electrophoresis to evaluate PP and selected TNP formulations, we observed equivalent protection from RNase (Figure S9) and similar gel retardation in the presence of high concentrations of heparin (>0.2 g/L; Figure S10). The low-throughput, qualitative, nonphysiological nature of gel electrophoresis^56^ motivated us to quantify RNA packaging using Cy3/Cy5 double-labeled saRNA, where tighter condensation results in higher FRET efficiency.^57^ We note that because bulk FRET measurements cannot discriminate between heterogeneous total RNA release *versus* homogeneous, modest loosening of the condensed RNA structure, we use the term ″loosening″ to describe a decrease in RNA packaging density throughout this manuscript to encompass both possibilities. As shown in Figure 2C, within the PEG_5k_-*bl*-polyanion_5k_ series, formulations containing polyanions with high charge density and hydrophobicity TNP5/8 (P5/P8 ∼ 278 *m*/*z* ^–^) and TNP6/9 (P6/P9 ∼ 185 *m*/*z* ^–^) showed higher FRET ratios (0.32) compared to the hydrophilic TNP1 (0.21) and naked saRNA (0.17) upon heparin challenge. Similarly, while increasing anionic block length (TNP28 to TNP31, TNP32 to TNP35) decreased FRET ratios in 1% FPBS buffer, incorporating higher hydrophobic content (TNP32 to TNP35) increased resistance to heparin-mediated loosening (FRET ratio 0.33 for TNP32 to TNP35 vs 0.25 for TNP28 to TNP31), consistent with our findings for the 5 kDa anion block series. This enhanced resistance to saRNA loosening provides further evidence that both electrostatic interactions and hydrophobic forces contribute to particle stability.

To further investigate the relationship between polyanion composition and surface properties, we performed a control experiment using mixtures of PEGylated and non-PEGylated polyanion_5k_ at a fixed C/N = 1 (Figure S11). For hydrophilic TNP2, only full (100%) PEG coverage achieved sufficient steric protection and maintained colloidal stability. In contrast, hydrophobic formulations TNP5 (25% BA) and TNP8 (50% BA) maintained stability even at reduced PEG densities of 75 and 50%, respectively, although the greatest size stability was still achieved with 100% PEG coverage. These findings further support our earlier observations that particle stability results from the interplay between PEG density and polyanion composition, where increased polyanion hydrophobicity can partially compensate for reduced PEG coverage in maintaining nanoparticle stability in salt and protein environments.

We next asked whether the PEG-polyanion properties endowing optimal size and packaging stability to pABOL-saRNA TNPs might also stabilize alternative nucleic acid cargoes or polycations. We found that pABOL-mRNA TNPs (838 nt, ∼268 kDa mRNA vs 9382 nt, ∼3 MDa saRNA) formulated at C/N = 1 with PEG_5k_-*bl*-polyanion_5k_ (TNP2/5/8) were similar in size to their saRNA counterparts (∼50 nm diameter) and that polyanion hydrophobicity again correlated with smaller TNP size in B-T formulation buffer (Figure S12). While mRNA TNPs with PEG_2k_-*bl*-polyanion_5k_ formed smaller particles at C/N = 1 than their saRNA counterparts (50 vs 100 nm diameter), PEG_2k_ was still insufficient to prevent salt- and protein-mediated aggregation. Thus, while a smaller cargo size may present less of barrier to polyanion remodelling of small TNPs, sufficient PEG length is ultimately required for steric stabilization. Plasmid DNA (pDNA; 4.6 kb, ∼1.4 MDa) PP were larger (73 vs 56 nm diameter) and contained some worm-like particles in contrast to highly spherical saRNA PP (Figure S13), which may be attributed to the lower flexibility of the double helix.^58^ Interestingly, only the coating polymer with the lowest charge density and hydrophobicity (P1) could form charge-neutralized pDNA-TNPs at C/N = 1 without fully unpackaging the pDNA cargo, implicating cargo flexibility as a determinant of TNP structural integrity. We then formulated PPs with either jetPEI or Polybrene and Cy3/Cy5-saRNA at N/P = 10 and TNPs using a variety of polyanions at C/N = 1 to evaluate the impact of polycation charge density, p*K*_a_, and hydrophobicity on TNP stability (Figure S14). Although PP with higher charge density polycations (jetPEI, Polybrene) were more tightly packaged than pABOL PP in B-T and PBS, their saRNA packaging decreased upon addition of coating polymers whereas that of pABOL TNPs increased. While jetPEI formed small TNPs with a wide range of polyanions, only TNP5 and TNP8 remained <250 nm in diameter when challenge with PBS ± 1% FBS. All Polybrene TNPs except TNP8 were >250 nm in diameter upon formulation. Collectively, these results demonstrate that nucleic acid flexibility and various polycation properties alter TNP formulation requirements, but that the PEG_5k_-*bl*-polyanion_5k_ architecture and pABOL-polyanion interactions synergize to stabilize TNPs.

### Small Angle Neutron Scattering Characterizes Environmentally Responsive Changes in saRNA TNP Shape and Surface Roughness

While previous studies have reported that polyanions improve PP stability,^29,31^ the structural transformations induced by polyanions have not been characterized and the mechanism of particle stabilization remains poorly understood. Building on our finding that polyanion charge density and hydrophobicity modulate TNP stability, we investigated their influence on the mesoscale structure of TNP in various buffer conditions. To correlate structural insights with stability profiles established by our DLS and FRET studies, we interrogated TNP morphology and mesoscopic structure with SANS experiments in a variety of physiological challenge buffers. This technique allowed us to characterize the compositional organization of PP and TNP, leveraging differences in particle vs solvent atomic scattering length densities (SLD) that result in particle-characteristic neutron scattering profiles (Table S2). While SANS has been used to study nucleic acid encapsulation in LNP^41,42^ and PP,^39^ to the best of our knowledge, its unique capability to probe nanoscale internal structures in solution state has not yet been applied to ternary polyelectrolyte nanoparticles.

We first applied shape-independent fitting approaches to compare PP and TNP surface roughness and shape in formulation buffer (Figure 3A and Table S3). For TNP1, as the Guinier region was not captured over this q range, we used a power-law model to fit the Porod region (*q* range: 0.00481–0.0181) to obtain the Porod exponent (Porod_exp) for comparison of surface roughness across formulations (Figure 3B and Table S3). Samples with a visible Guinier region were fit with a Guinier-Porod model over a wider *q* range to additionally obtain sphericity (*s*) and radius of gyration (*R*_g_) parameters (Table S3). In agreement with transmission electron microscopy (TEM) observations (Figure 3C), Guinier-Porod fitting found protonated PP to be highly spherical (*s* = 0.00 ± 0.04) with a rough surface (Porod_exp = 3.6 ± 0.4) with *R*_g_ = 20.8 ± 0.6 nm, which is slightly smaller than the hydrodynamic radius (*R*_h_) measured by DLS (∼28.0 nm). A difference between the *R*_g_ and *R*_h_ is expected because neutron scattering is proportional to differences in nuclear density and the scattering length per unit volume, whereas light scattering is proportional to differences in refractive index and includes contributions from surface-associated solvation shells. The TNP with lowest anionic charge density (TNP1) exhibited a Porod_exp in the mass fractals regime which can represent scattering from Gaussian polymer chains or two-dimensional structures^59^ (Porod_exp = 1.9 ± 0.1), which reflects the larger amount of neutral hydroxyethyl acrylate (HEA) present in this formulation that does not participate in electrostatic condensation. All other TNP displayed smooth surfaces (Porod_exp 4.1–4.5) with altered shape features (*s* = 0.49–0.68), the latter of which may be the result of shape changes or polydispersity inherent in self-assembled structures not captured by this simple shape-independent model. Indeed, dry-state TEM images showed that all TNP exhibited spherical morphologies, with high charge density (TNP3) and hydrophobicity (TNP8) formulations displaying well-defined core–shell structures (Figure 3C). Because excess polycation (N:P > 1) is required to form PP and could theoretically form polyion complexes (PIC) with polyanions, we performed dual-color nano flow cytometry (nanoFCM) to assess the formation of RNA cargo-less PICs (Figure S15). We observed that 98.5% of AF488-P5^+^ particles were Cy5-saRNA^+^ (gated on the intensity of Cy5-saRNA^+^ PP), with only 1.5% Cy5-saRNA^–^ empty PIC in the TNP sample. While nanoFCM approaches single AlexaFluor per nanoparticle detection sensitivity,^60^ it cannot quantify unbound polymer and may miss very small PICs. Complementary measurements with Fluorescence Cross-Correlation Spectroscopy^61^ or Single Particle Automated Raman Trapping Analysis^62^ would help further define TNP composition and mechanisms of self-assembly. Taken together, our EM and nanoFCM data imply that TNPs and not empty particles dominate the scattering profiles in the DLS and SANS data presented.

**Figure 3:**
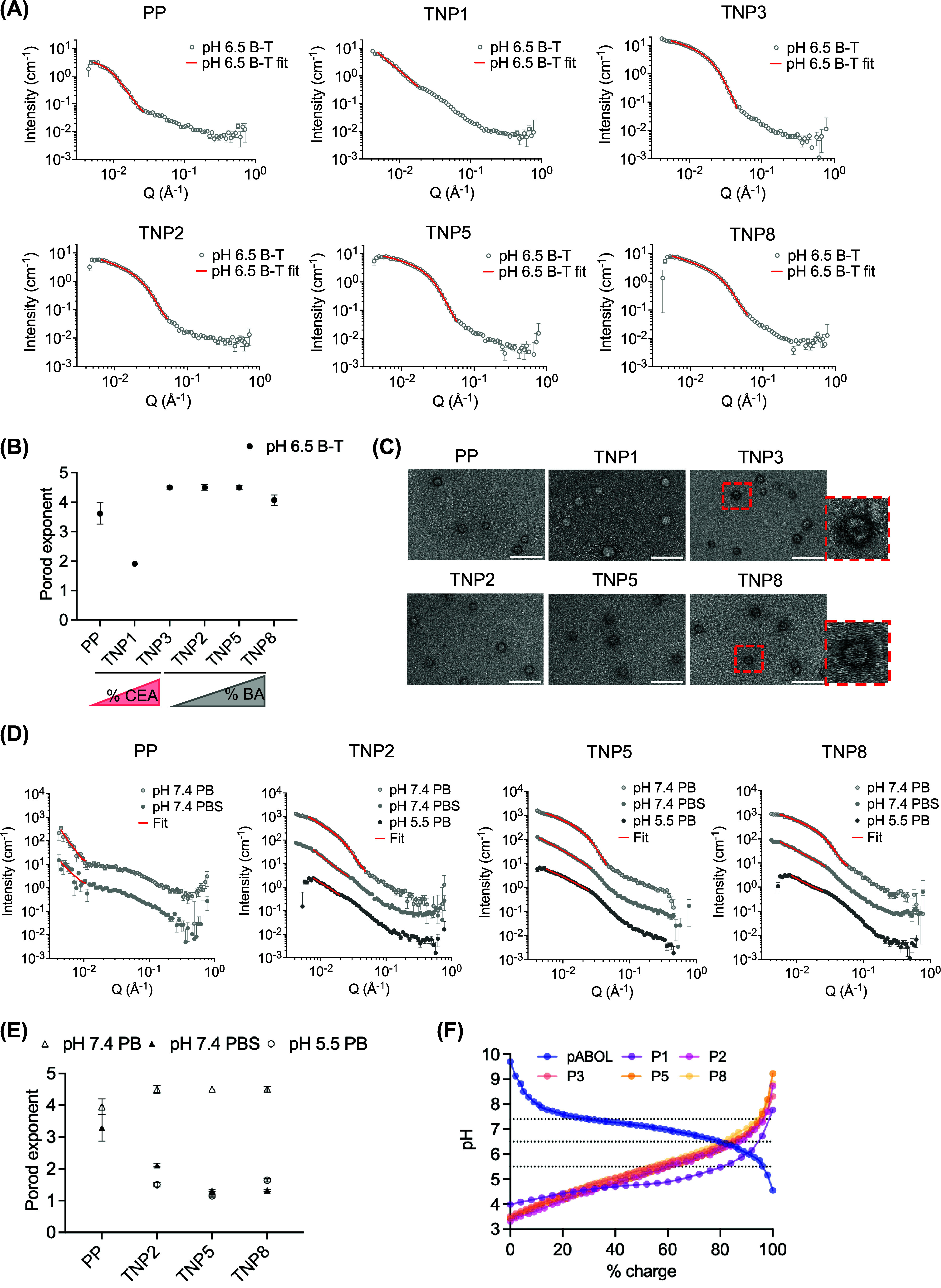
Ternary nanoparticles exhibit pH-responsive morphological changes. (A) SANS data of PP and TNP in pH 6.5 Bis-Tris (B-T) and Guinier-Porod fits (TNP1 was fitted with power-law model). (B) Porod exponents and fitting errors. (C) TEM images of PP and TNP. Scale bar = 200 nm. (D) SANS data of PP and TNP in various buffer conditions. Samples in pH 7.4 PB were fitted with the Guinier-Porod model, the rest with the power-law model. Data and fit curves of samples pH 7.4 PB and pH 7.4 PBS are offset by factors of 100 and 10, respectively, for better visualization. (E) Porod exponents and fitting errors. (F) Polyelectrolyte charge titrations; the physiological pH range studied (5.5–7.4) is outlined with dotted lines.

We further characterized the impact of coating polymer hydrophobicity on TNP surface roughness and shape in a variety of physiological challenge buffers (Figure 3D,3E and Table S3). Data for TNP in pH 7.4 PB were fitted using the Guinier-Porod model, while power-law model was applied for all other samples due to their lack of a clear Guinier region. As expected, PP aggregated in buffers with high pH or ionic strength where pABOL is deprotonated, evidenced by an extreme upturn in scattering intensity at low-*q* region. In contrast, TNP exhibited buffer-dependent structural changes without aggregating. At pH 7.4 in the absence of NaCl, all TNP possessed similar smooth surfaces (Porod_exp = 4.5 ± 0.1), with reduced sphericity (*s* = 0.84–1.33 vs *s* = 0.49–0.68 in pH 6.5 Bis-Tris). Based on the p*K*_a_ of the polyelectrolytes present, this is likely the result of electrostatic repulsion between saRNA and coating polymers following polycation deprotonation (Figure 3F). In physiological saline (pH 7.4 PBS), all TNP exhibited decreased Porod_exp between 1.3 and 2.1, suggesting a morphology typical of flexible, loose polymer chains, attributed to electrostatic screening among RNA, pABOL, and polyanions. Interestingly, at pH 5.5 (representative of the late endosome and close to coating polymer p*K*_a_), all three TNP exhibited similar surface roughness as in pH 7.4 PBS. Coating polymer protonation at acidic pH (Figure 3F) could plausibly increase hydrophobicity while decreasing coating polymer-polycation electrostatic affinity, releasing amphipathic polyanions that are known to facilitate endosomal escape.^53^ This pH-dependent release mechanism aligns with our hemolysis data, where P8 demonstrated selective membrane-disruptive activity at pH 5.5 (Figure S8). These results provide initial evidence of how polyanion properties influence TNP organization; however, a deeper understanding of the internal mesoscopic structure requires a more sophisticated shape-dependent model capable of distinguishing between different polymeric domains.

### Polyanion Hydrophobicity Offsets pH-Responsive Swelling of TNP Core–Shell Structures

Inspired by our FRET data, TEM images, and shape-independent SANS fitting results, we hypothesized that SANS might distinguish between polymeric domains condensing RNA in the core and those at the solvent interface. We thus applied the core_shell_sphere model to quantify particle structure and SLD compositions with low fitting error (χ^2^; Figure 4A–C and Table S4). To aid interpretation of fitting results, we have represented calculated SLD values using a color-coded scale^41^ (Figure 4B and Table S2) and have included schematics depicting relative core/shell size and SLD (Figure 4C). Because PEG is extremely flexible and associated with bound D_2_O, its neutron scattering contrast is minimal.^41^ We instead posit that neutron contrast is dominated by the scattering of the core and shell domains of tightly (SLD × 10^6^ ≈ 1.00 Å^–2^) and loosely (SLD × 10^6^ ≈ 6.00 Å^–2^) packed polyelectrolytes with different hydrations, respectively. In formulation buffer where all polyelectrolytes are highly charged, we found that PP consist of a tightly condensed core (SLD × 10^6^ = 1.23 ± 0.51 Å^–2^; radius = 3.3 ± 0.2 nm) and a larger, hydrated shell (SLD × 10^6^ = 6.12 ± 0.02 Å^–2^; thickness = 17.2 ± 0.3 nm), whereas TNP core/shell domain size and hydration correlated with polyanion chemistry. Polyanions with high negative charge density (P3; SLD × 10^6^ = 2.18 ± 0.22 Å^–2^; radius = 9.1 ± 0.0 nm) promoted TNP core hydration and expansion relative to low charge density polyanions (SLD × 10^6^ = 1.37 ± 0.22 Å^–2^ and radius = 7.4 ± 0.1 nm for P2), with an expanded shell (thickness = 19.1 ± 0.7 nm). Conversely, hydrophobic polyanions (P8) resulted in a denser core and thinner shell (radius = 6.3 ± 0.0 nm and thickness = 14.6 ± 0.4 nm) compared to its hydrophilic counterparts with equal charge density (radius = 7.4 ± 0.1 nm and thickness = 15.2 ± 0.6 nm for P2; radius = 7.4 ± 0.0 nm and thickness = 16.0 ± 0.5 nm for P5), indicating that hydrophobic interactions can promote tighter packing between polyanion/polycation chains. Together, we conclude that differences in polyanion charge density and hydrophobicity modulate the hydration and compactness of the TNP core, as well as the thickness of the shell. To demonstrate the significance of a three- *versus* two-component polyelectrolyte formulation, we benchmarked these structural changes against PP formed with a PEGylated pABOL (PEG–PP). We found that PEG–PP exhibited a more hydrated core (SLD × 10^6^ = 1.71 ± 0.31 Å^–2^) compared with uncoated PP or TNP coated with low charge density polyanions (TNP2/P5/P8). This observation can be attributed to the presence of covalently attached PEG chains, which introduce steric hindrance that may interfere with RNA packaging during the formation of PEG–PP.

**Figure 4:**
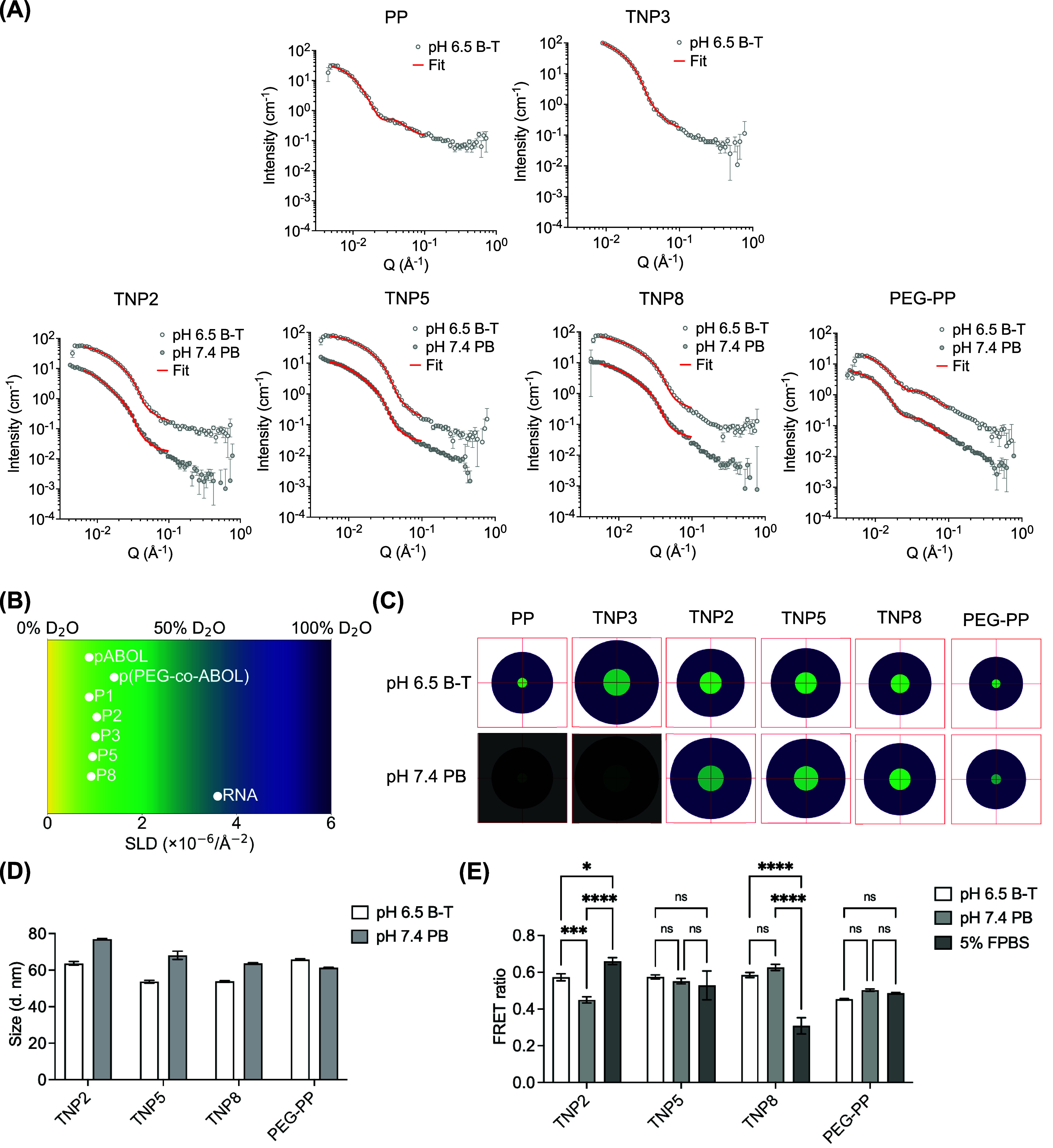
Polyanion chemistry modulate TNP core–shell structural responses. (A) SANS data were fitted with the core_shell_sphere model. Data and fit curves of samples pH 7.4 PB are offset by a factor of 10 for better visualization. (B) A color-coded SLD scale was applied for SANS analysis. (C) 2D projections of the core–shell domain sizes and SLDs. Red square = 60 nm × 60 nm. (D) DLS measurements of Z-average hydrodynamic diameter (intensity-weighted). (E) FRET analysis of RNA packaging in different buffer conditions. Data shown as mean ± SD, *N* = 3. Statistical significance was calculated using one-way analysis of variance (ANOVA) and Tukey’s multiple comparisons. ****p < 0.0001, ***p < 0.001, *p < 0.05, ns means not significant.

We next studied TNP2/P5/P8 and PEG–PP at pH 7.4 to elucidate how polyanion hydrophobicity influences the core–shell structure in the absence of polycation protonation (Figure 4C and Table S4). All TNP retained overall colloidal stability characterized by DLS (Figures 4D and S16), with underlying structural differences dependent on coating polymer chemistry. In saline-free conditions at pH 7.4, TNP exhibited increases in core radius/SLD and shell thickness indicative of swelling. However, TNP coated with hydrophobic polyanions (TNP5/P8) exhibited significantly smaller changes in core SLD compared to TNP2, confirming the role of hydrophobicity in enhancing structural integrity against pH-induced destabilization. PEG–PP exhibited a more hydrated core (SLD × 10^6^ = 2.53 ± 0.34 Å^–2^) than TNP, aligning with prior suggestions that direct polycation PEGylation may increase PP susceptibility to unpackaging.^19^

The FRET RNA packaging data echoed the environmentally responsive structural changes observed by SANS (Figure 4E). At pH 7.4, TNP2 exhibited the lowest FRET ratio, suggesting a less condensed core, while hydrophobic coating polymers (P5/P8) mitigated TNP pH-dependent FRET decrease. However, TNP8, featuring the highest hydrophobicity, exhibited compromised RNA packaging stability in serum-containing conditions, revealing a critical trade-off between hydrophobic stabilization and serum protein interactions. In contrast to TNP, PEG–PP demonstrated no pH-responsive FRET signal. These findings illustrate how supramolecularly PEGylating polyplexes with PEG-polyanions also alters nanoparticle structural stability from the inside-out. In particular, balancing charge density and hydrophobicity (TNP5) offers superior stability at higher pH, ionic strength, and protein concentration. Furthermore, by validating SANS-derived structural data with DLS and FRET measurements, we also position these high-throughput assessments of nanoparticle structural stability to accelerate future formulation development.

### Polyanion Chemistry Alters TNP Cellular Internalization and saRNA Transfection

We investigated how TNP structural differences influence saRNA delivery in DC2.4 cells, a cell line representative of putative vaccine targets, by evaluating cellular uptake (Figures 5A,5B, and S17) and transfection efficiency (Figure 5C). Importantly, polyanion coating did not increase cytotoxicity compared to uncoated PP, with all formulations maintaining cell viability around or above 80% regardless of media conditions (Figure S18).

**Figure 5:**
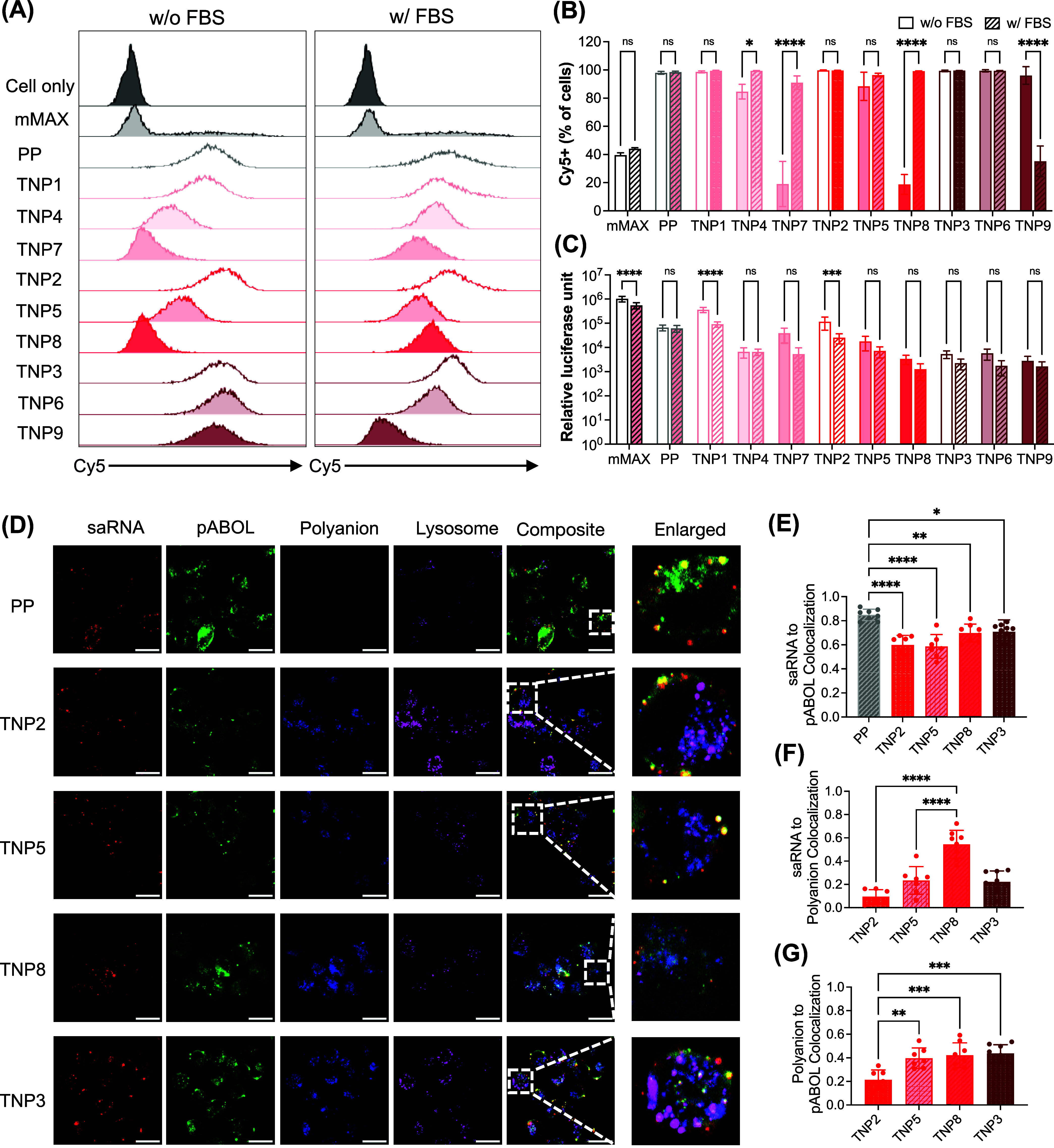
Polyanion chemistry alters TNP cellular internalization and saRNA transfection. (A) Representative flow cytometry histograms and (B) quantification of Cy5-saRNA+ DC2.4 cells. Data shown as mean ± SD, *N* = 3. (C) Transfection efficiency of firefly luciferase (fLuc) saRNA in DC2.4 cells. Data shown as mean ± SD, *N* = 3. (D) Representative confocal microscopy images of DC2.4 cells treated with PP or TNP containing Cy5-labeled saRNA, FITC-pABOL, and AZDye405-polyanions for 1 h in the presence of FBS. The acidic compartments were stained with Lysotracker Red. Scale bar = 20 μm. Manders’ overlap coefficients of colocalization of (E) Cy5-saRNA and FITC-pABOL, (F) Cy5-saRNA and AZDye405-polyanion, (G) AZDye405-polyanion and FITC-pABOL. Data shown as mean ± SD, *n* > 7. Statistical significance in (B, C, E–G) was calculated using one-way analysis of variance (ANOVA) and Tukey’s multiple comparisons. ****p < 0.0001, ***p < 0.001, **p < 0.01, *p < 0.05, ns means not significant.

The commercial control lipofectamine messengerMAX (mMAX) exhibited low cellular uptake (∼40%) due to aggregate formation in culture media, yet achieved superior transfection efficiency, demonstrating that aggregation does not preclude effective delivery when intracellular processing is optimized. Under serum-free conditions, polyanion properties significantly influenced intracellular uptake and transfection. In the low charge density TNP series (25% CEA, ∼555 *m*/*z* ^–^; or 50% CEA, ∼278 *m*/*z* ^–^), increasing hydrophobicity led to a noticeable decrease in cellular uptake and correspondingly reduced transfection efficiency. While the high charge density series (75% CEA, ∼185 *m*/*z* ^–^) retained efficient uptake regardless of hydrophobicity, transfection efficiency was consistently low, suggesting that high density charge interactions may interfere with intracellular processing. Notably, TNP1 achieved the highest transfection efficiency despite having the worst colloidal stability, indicating that aggregating TNPs may be useful for local delivery.^21^ This phenomenon parallels our observation that aggregating mMAX/saRNA achieved superior transfection despite poor colloidal stability.

Serum-containing media significantly enhanced the uptake of hydrophobic TNP (TNP4/P7/P8), which could be attributed to receptor-mediated uptake due to serum protein adsorption on hydrophobic coating polymers.^63^ This protein corona hypothesis is consistent with our FRET data correlating coating polymer hydrophobicity with increased RNA loosening in protein-containing media (Figure 4E). However, protein-enhanced uptake did not translate to improved transfection, suggesting that protein adsorption may affect both uptake and downstream processing. Interestingly, TNP9, combining both high charge density (75% CEA) and high hydrophobicity (25% BA), showed an opposite trend with dramatically reduced serum uptake and no change in transfection efficiency. Thus, while polyanion hydrophobicity generally enhances serum-mediated uptake, the simultaneous increase in both charge density and hydrophobicity can disrupt this pattern, highlighting how nanomaterial properties cooperatively determine cellular interactions.

To investigate the discrepancy between uptake and transfection, we assessed the intracellular processing of TNP using confocal laser scanning microscopy (CLSM) (Figures 5D–G and S19–S21). After 1 h treatment in the presence of serum, both PP and TNP formulations were internalized effectively by DC2.4 cells (Figure 5D). All formulations resulted in low Cy5-saRNA colocalization with lysosomes (Manders’ coefficient <0.1), indicating successful avoidance of lysosomal degradation. However, saRNA was observed primarily in punctate compartments throughout the cytoplasm, with some diffuse distribution, suggesting that saRNA was retained in other endosomal compartments. Complementary assays^64^ are needed to definitively characterize the extent of endosomal escape and intracellular fate of the delivered saRNA. However, saRNA release from the pABOL core varied across formulations. Colocalization analysis between Cy5-saRNA and FITC-pABOL revealed that PP demonstrated significantly higher saRNA-pABOL association compared to TNP, indicating more restricted RNA release in uncoated formulations. Among the TNP, we observed a trend of reduced saRNA-pABOL association in TNP coated with P2/P5 vs their hydrophobic counterpart P8, and in TNP coated with P2 vs its high charge density counterpart P3, though statistical significance was not achieved (Figure 5E). In contrast, TNP8 showed significantly higher saRNA-polyanion colocalization compared to TNP2/5 (Figure 5F), suggesting either reduced polyanion detachment from the pABOL core (Figure 5G) or unexpected persistent saRNA-polyanion interactions impeding RNA cytoplasmic functionality. In serum-free conditions, increasing polyanion hydrophobicity (from TNP2 to TNP5 and TNP8) correlated with diminished intracellular Cy5-saRNA (Figure S19A), confirming flow cytometry results. The high colocalization of saRNA-polyanion observed for both highly hydrophobic (TNP8) and high charge density (TNP3) TNP further supports the hypothesis that these formulations trap saRNA in stable complexes, preventing efficient release and transfection (Figure S19D). Because pABOL is mostly degraded by 4 h (Figures S20 and S21), rapid polyanion shedding before this time point is essential for transfection. Taken together, these discrepancies between uptake and transfection in serum demonstrate that balancing charge density and hydrophobicity is essential to endow TNP with structural stability without sacrificing intracellular responsiveness, echoing past findings with hydrophobic cationic materials.^65–67^ Finding that greater TNP butyl acrylate content significantly decreased RNA packaging tightness (Figure 4E) but also inhibited TNP disassembly (Figure 4F) in the presence of serum prompted us to investigate the molecular structures that could drive the formation of a tightly bound protein corona.

### Molecular Dynamics Modeling Reveals Structural Drivers of Biological Performance

We used MD simulations to model the chemical and environmental factors governing TNP self-assembly and function (Figure 6). A coarse-grain (CG) MARTINI force field model (Figures S22–S24) enabled the analysis of minimalist PP and TNP assemblies (Figure 6A) and their structural stability across 50 ns in physiologically relevant buffers.^68,69^ Buffer conditions were simulated by increasing ionic strength and altering the percent of ionized polymer beads for each pH according to the polyelectrolyte p*K*_a_ and the Henderson–Hasselbach equation (Table S5 and Table S6).

**Figure 6:**
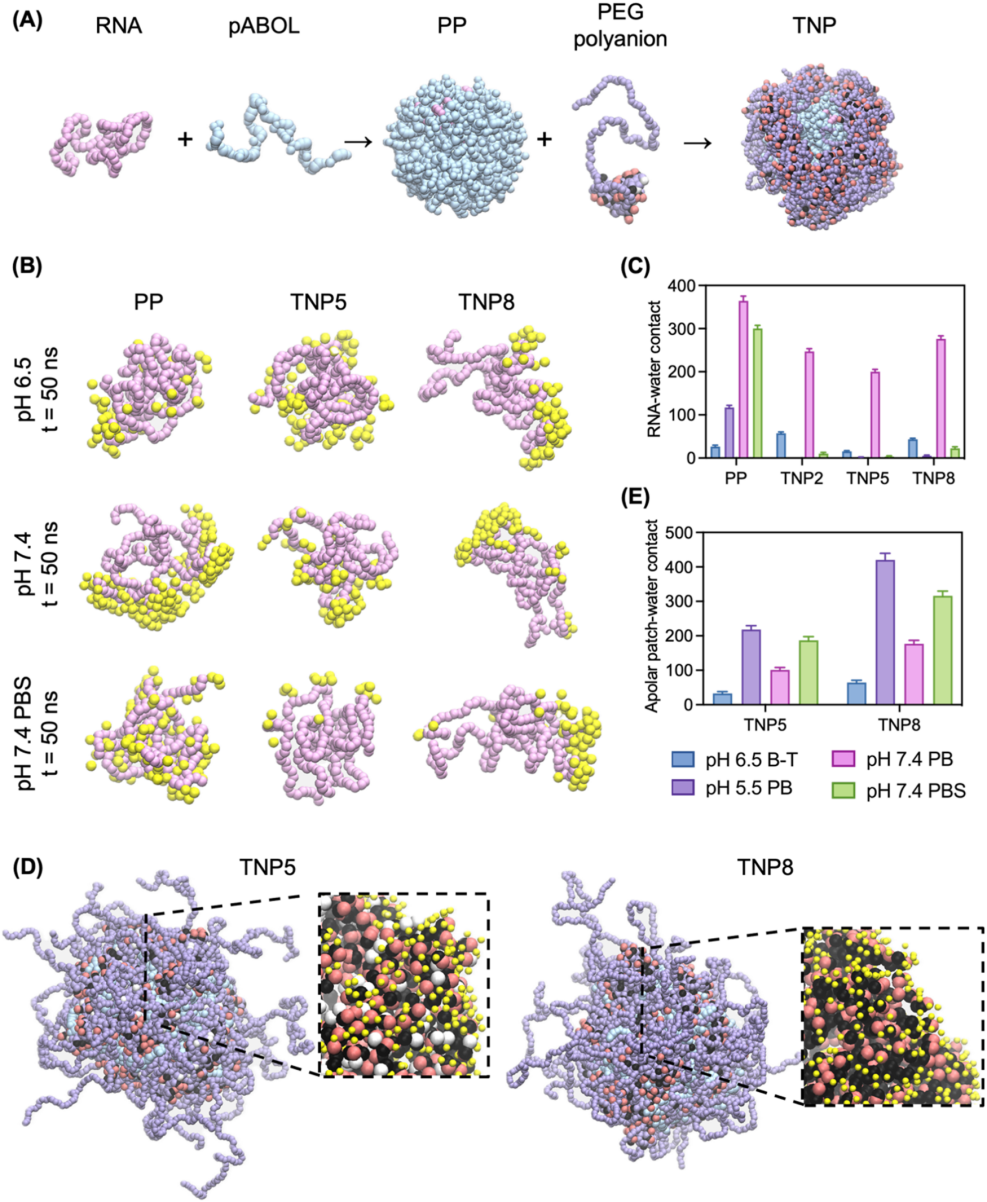
Coarse-grain molecular dynamics provide insight into mechanisms of RNA protection and protein binding. (A) Representative initial conformations of macromolecules simulated (pink = RNA; blue = pABOL; purple = PEG; black = butyl side chain; red = carboxyethyl side chain; white = hydroxyethyl side chain). (B) Snapshots of water (yellow) within 1 nm of RNA (pink) after 50 ns simulation in various buffers. (C) Quantification of the number of contacts between RNA and water in various formulations during 50 ns simulation in various buffers. (D) Snapshots of TNP after 50 ns simulation in pH 7.4 PBS, with insets showing apolar surface residues (black) exposed to water (yellow). (E) Quantification of the number of contacts between TNP5 and TNP8 apolar residues and water during 50 ns trajectories in various buffers. Data shown as mean ± SD averaged across the last 2 ns in the simulation trajectory.

To understand the SANS finding that coating polymer chemistry modulates core hydration, we first investigated RNA-water contacts in PP and TNP in various buffers. Simulation snapshots showed that water molecules (yellow) were able to access RNA (pink) in both PP and TNP, with this effect particularly pronounced at pH 7.4 (Figure 6B). Quantitative analysis revealed that PP experienced the most dramatic increase in RNA-water contacts when pABOL was deprotonated at pH 7.4, indicating significant water penetration and potential destabilization at physiological pH (Figure 6C). While all TNP also showed increased water penetration at pH 7.4 (corroborating our SANS findings), they maintained better RNA protection than uncoated PP. In PBS conditions, TNP demonstrated superior RNA shielding from water compared to uncoated PP (Figure 6B,C), indicating that polyanion coating prevents core swelling when electrostatic forces are screened. These simulations provide molecular-level insights into SANS characterization of how polyanions modulate TNP core hydration.

Trajectory analysis further revealed the role of butyl acrylate residues at the TNP5/P8 particle surface. The number of apolar patch-water contacts varied with buffer conditions (Figure 6D,E). For example, butyl groups were nearly completely buried at pH 6.5 (polycation 80% charged, polyanion 93–96% charged) but were exposed to water at pH 5.5 (polycation 100% charged, polyanion 57–70% charged) or pH 7.4 (polycation 33% charged, polyanion >99% charged). After 50 ns simulation in PBS, hydrophobic residues in TNP5/P8 formulations transitioned from their initially shielded core positions to increased water exposure (Figure 6D) and the amount of butyl acrylate per polymer correlated with the amount exposed (Figure 6E). This discovery of solvent-exposed hydrophobic residues suggests a mechanism of protein adsorption to TNP8 that correlates with our observations of serum-induced cellular uptake (Figure 5) and compromised RNA packaging tightness (Figure 4), underscoring the role of coating polymer chemistry in altering formulation function from the inside-out. These results again highlight how balanced polyanion properties maintain particle structural integrity while modulating protein-binding.

## Conclusion

In summary, we have used a combination of high- and low-throughput synthetic and experimental workflows to systematically decipher how polyanion chemistry influences ternary polyelectrolyte nanoparticle structure and function. By synthesizing a library of chemically diverse polyanions, we demonstrated that a diblock PEG-polyanion architecture with equal molecular weights of each block and optimized balance of anionic charge density and hydrophobicity are essential for colloidally stable, charge-neutralized TNPs. Hydrophobic polyanions resulted in TNPs with compact cores and thin shells, while hydrophilic and highly charged variants promoted core hydration and shell expansion. Notably, while all TNPs demonstrated pH-responsive structural changes in pH 7.4 dominated by polycation deprotonation, hydrophobic polyanions limited increases in TNP core size and hydration. Hydrophobicity-enhanced stability observed by DLS, FRET, and SANS correlated with reduced uptake in serum free conditions, underscoring how stabilizing polyanions can sterically shield nonspecific PP uptake mechanisms. However, excessive polyanion hydrophobicity drove serum-dependent uptake and limited intracellular release and saRNA expression, implying that polyanion hydrophobicity modulates TNP protein binding. Indeed, MD simulations confirmed that polyanions effectively shield RNA in the core from water and revealed solvent-accessible apolar surfaces that could explain the observed protein-dependent behavior. These computational and experimental insights establish a framework for understanding the balance required in polyanion design: sufficient hydrophobicity for enhanced stability balanced against excessive protein binding and reduced functionality. Because we found that TNP stability is largely governed by polycation p*K*_a_, future work should employ polycations with higher p*K*_a_ and greater hydrophobicity than pABOL to maximize interactions with stabilizing polyanions. Further engineering of polyanion p*K*_a_, hydrophobicity, and block length/architecture could be used to modulate protein binding or facilitate cooperative deprotonation for rapid TNP disassembly. Evaluating next generation TNP formulations *in vivo* will be critical to demonstrate the translational potential of our screening approach. The connections we identify between polymer chemistry, mesoscale structure, and molecular modeling reveal the pH-responsive nature of structure/function in TNP systems at the nanobiological interface and provide a mechanistic roadmap to engineer RNA delivery vehicles with dynamic stability.

## Materials and Methods

### Materials

2-Carboxyethyl acrylate (CEA), 2-hydroxyethyl acrylate (HEA), butyl acrylate (BA), poly­(ethylene glycol) methyl ether acrylate (OEGA, average Mn 480) were purchased from Sigma-Aldrich and deinhibited using inhibitor removal resin prior to use. 2-(*n*-butyltrithiocarbonate)-propionic acid (BTPA) was purchased from Boron Molecular. Poly­(ethylene glycol) methyl ether (PEG_2k_ and PEG_5k_, average Mn 2000 and 5000) and azide poly­(ethylene glycol) (N_3_–PEG_5k_, average Mn 5000) were purchased from Sigma-Aldrich and Biopharma PEG, respectively. *In vivo*-jetPEI was purchased from Polyplus-Sartorius. Polybrene, 4-(dimethylamino)­pyridine (DMAP), *N*,*N*′-dicyclohexylcarbodiimide (DCC), 5,10,15,20-tetraphenyl-21*H*,23*H*-porphine zinc (ZnTPP), 3-(trimethylsilyl)-1-propanesulfonic acid sodium salt (DSS), deuterated dimethyl sulfoxide (DMSO-*d*_6_), dimethyl sulfoxide (DMSO), dichloromethane (DCM), and diethyl ether were purchased from Sigma-Aldrich. AZDye 405 DBCO was purchased from Vector Laboratories. PureLink HiPure plasmid filter maxiprep kit (Invitrogen, Thermo Fisher Scientific), pGL4.5 CMV-Luc vector (ProMega), MluI-HF (New England Biolabs), HiScribe T7 high yield RNA synthesis kit (New England Biolabs), Cy3-UTP (ApexBio), Cy5-UTP (ApexBio), and ScriptCap Cap 1 capping system (Cambio) were used for nucleic acid preparation. Cell culture materials included RPMI 1640 medium, Opti-MEM, fetal bovine serum (FBS), penicillin-streptomycin, HEPES (all from Gibco, Thermo Fisher Scientific), 2-mercaptoethanol (Sigma-Aldrich), MEM nonessential amino acid solution (100×, Sigma-Aldrich), ONE-Glo luciferase assay system (Promega), cell counting kit 8 (CCK-8, Abcam), Pierce 16% formaldehyde (w/v) methanol-free (PFA, Thermo Fisher Scientific), lysotracker red DND-99 (Invitrogen, Thermo Fisher Scientific), lipofectamine messengerMAX transfection reagent (Thermo Fisher Scientific). All reagents were used as received unless otherwise specified.

### pABOL Synthesis

poly­(CBA-*co*-ABOL) “pABOL” was synthesized by aza-Michael polyaddition of 4-amino-1-butanol (ABOL; 1 equiv) to N,N’-cystaminebis­(acrylamide) (CBA; 1.004 equiv) reacted for 12 days and purified by dialysis and lyophilization according to the previously published protocol.^44^ Polymer identity was confirmed by ^1^H NMR and organic GPC analysis confirmed *M*_n_ = 11.2, *M*_w_ = 26.2 kDa, and *Đ* = 2.33 (Figure S25).

### FITC-pABOL Synthesis

pABOL (50 mg) and triethylamine (20 μL) were dissolved in DMF (2 mL) with stirring. Fluorescein isothiocyanate (FITC) (1 mg in 100 μL DMF) was added and mixed in the dark at 25 °C for 24 h. The reaction was then purified by dialysis and lyophilization according to the previously published protocol.^44^

### p­(PEG_5k_-*co*-ABOL) Synthesis

p­(PEG_5k_-*co*-ABOL) was synthesized in the same manner as pABOL but with 1.004 eq CBA, 0.95 eq ABOL, and 0.05 eq mPEG_5k_-NH_2_ (BioPharma PEG; MF001005–5K) reacted for 12 days and dialyzed against acidified water (pH 4) in 10 kDa Snakeskin dialysis tubing for 3 days before lyophilization. Organic GPC analysis confirmed *M*_n_ = 15.7, *M*_w_ = 24.6 kDa, and Đ = 1.562; DOSY NMR confirmed the absence of nonpolymerized PEG and ^1^H NMR quantified 1.94 PEG and 12.66 ABOL side chains per polymer (Figure S26).

### Polyanion Synthesis

#### Synthesis of PEGylated Macro-CTAs

PEGylated 2-(*n*-butyltrithiocarbonate)-propionic acid (PEG-BTPA) was synthesized *via* an esterification reaction following established literature procedures.^70^ BTPA (4.2 mmol, 1 g) was dissolved in dry dichloromethane (DCM, 30 mL) and added to a 100 mL round-bottom flask containing PEG (2.1 mmol; 10.487 g for PEG_5k_ or 4.195 g for PEG_2k_) and a magnetic stir bar. A solution of 4-(dimethylamino)­pyridine (DMAP, 0.42 mmol, 0.051 g) and N,N’-dicyclohexylcarbodiimide (DCC, 4.2 mmol, 0.866 g) in dry DCM (15 mL) was added dropwise at 0 °C. The flask was sealed with a rubber septum and purged with nitrogen for 30 min. The reaction mixture was then brought to room temperature and stirred for 48 h. Precipitated dicyclohexylurea was removed by filtration through a 2–3 μm filter paper. The DCM layer was precipitated twice in cold diethyl ether, and the resulting pellet was dried overnight under vacuum, yielding PEG_5k_-BTPA and PEG_2k_-BTPA macro-CTAs.

#### Synthesis of Fluorophore-Conjugated Macro-CTAs

Azide-functionalized PEGylated macro-CTAs were synthesized following the above procedure, substituting N_3_–PEG_5k_ for PEG. The purified N_3_–PEG_5k_-BTPA was dissolved in DMSO (0.05 M) and functionalized with AZDye 405 DBCO (0.05 M in DMSO) through a strain-promoted azide–alkyne cycloaddition reaction in equimolar ratios. The resulting AZDye405-PEG_5k_-BTPA was stored in DMSO at a concentration of 0.25 M.

#### Polyanion Library Synthesis

Polyanions were synthesized using PET-RAFT polymerization,^71,72^ initiated from either BTPA or PEG-BTPA macro-CTAs. Monomers included 2-carboxyethyl acrylate (CEA), 2-hydroxyethyl acrylate (HEA), butyl acrylate (BA), and poly­(ethylene glycol) methyl ether acrylate (OEGA). 5,10,15,20-tetraphenyl-21*H*,23*H*-porphine zinc (ZnTPP) was employed as the photocatalyst, and 3-(trimethylsilyl)-1-propanesulfonic acid sodium salt (DSS) was used as an internal standard for ^1^H NMR. Stock solutions in DMSO were prepared for multiwell plate polymerization, including CTA (0.05 M), ZnTPP (0.01 M), monomers (CEA, HEA, BA at 3 M and OEGA at 2.3 M), and DSS (0.05 M). Components were mixed according to designed compositions with 1 M total monomer concentration and ZnTPP/CTA = 0.01:1 (m/m, for 5 kDa anion block) or 0.02:1 (m/m, for 10–40 kDa anion block). A typical synthesis of mPEG_113_-*bl*-p­(CEA_20_-*st*-HEA_10_-*st*-BA_10_) block copolymer is presented. The reaction mixture was composed of PEG_5k_-BTPA (60 μL, 0.05 M), CEA (20 μL, 3 M), HEA (10 μL, 3 M), BA (10 μL, 3 M), ZnTPP (3 μL, 0.01 M), and DSS (17 μL, 0.05 M) to make up a 120 μL reaction mixture (20 μL reserved for t_0_^1^H NMR). The remaining 100 μL prepared reaction mixture was pipetted to 96-well half area plates (100 μL reaction volume with 20 μL of mineral oil sealant). Polymerizations proceeded at room temperature for 6 h under yellow LED irradiation (λ = 590 nm), achieving monomer conversions >90% (P1–P27, 5 kDa anion block polyanions) as confirmed by ^1^H NMR to minimize potential effects of residual monomer on formulation and cellular interactions.

#### Fluorophore-Conjugated Polyanion Synthesis

10% AZDye405-conjugated polyanions were polymerized from a mixture of AZDye405-PEG_5k_-BTPA and PEG_5k_-BTPA in a molar ratio of 0.1:1.^73^ Polymerizations were conducted following the above procedure, but on a small scale in 384-well plates with 35 μL reaction volumes.

#### Polymer Characterization

All ^1^H NMR spectra were recorded using a Bruker Avance III HD 600 MHz spectrometer. Measurements were conducted in deuterated dimethyl sulfoxide (DMSO-*d*_6_), with chemical shifts internally referenced to residual solvent resonances. Monomer conversions were determined by comparing the intensity of unreacted acrylate vinyl peaks to the DSS internal standard.

#### Gel Permeation Chromatography (GPC)

Polyanion molecular weight (*M*_n_, *M*_w_) and polydispersity (Đ) were analyzed using aqueous GPC on a Viscotek GPC/TDAmax system. The system was equipped with an Agilent PL aquagel–OH guard column (50 mm × 7.5 mm, 8 μm), and two Agilent PL aquagel–OH analytical columns: OH 30 (300 mm × 7.5 mm, 8 μm) and OH 40 (300 mm × 7.5 mm, 8 μm), connected in series. The mobile phase comprised 0.2 M sodium nitrate (NaNO_3_) and 0.01 M phosphate buffer (PB) in water (pH 7.4) and 10% v/v methanol. The flow rate was set at 1 mL/min. Molecular weights were calibrated using refractive index detection and near-monodisperse poly­(ethylene oxide) (PEO) standards.

Polycations (pABOL and p­(PEG_5k_-*co*-ABOL)) were characterized following methods reported previously.^44^ Specifically, GPC analysis was performed using an Agilent PL GPC-50 instrument equipped with a refractive index (RI) detector running in HPLC grade DMF (containing 0.075 wt % LiBr) at a flow rate of 1.0 mL/min at 40 °C through two GRAM Linear columns (Polymer Standards Service) in series. Near-monodisperse poly­(methyl methacrylate) standards dissolved in eluent were used to calibrate the instrument. Polymer was dissolved in eluent at 2 mg/mL and filtered through a 0.2 μm syringe filters prior to analysis.

#### p*K*_a_ Measurement

Polymer acid–base titrations were performed using an EasyMax 102 reactor (Mettler Toledo) equipped with a pH probe, according to previously published procedures.^50^ For polycation titration, pABOL or p­(PEG-*co*-ABOL) was dissolved in 0.05 M NaCl to achieve a final polymer concentration of 4 mg/mL. The pH was adjusted to 2 by adding 2 M HCl to fully protonate the amine groups. The solution was then titrated with 0.2 M NaOH at a rate of 0.05 mL/min while continuously monitoring the pH until it reached 12. For polyanion titration, polyanion was dissolved in 0.05 M NaCl (4 mg/mL final concentration), adjusted to pH 12 by adding 2 M NaOH to fully deprotonate the carboxyl groups, then titrated with 0.2 M HCl (0.05 mL/min) until pH decreased to 2. The p*K*_a_ value was determined as the pH at the half-equivalence point of the titration curve, using the first derivative of the pH *versus* volume to identify equivalence points. The degree of deprotonation or protonation (θ) was converted by setting a value of 0% to the first equivalence point and a value of 100% to the second equivalence point. Hill coefficients (*n*_Hill_) was estimated from the linear range of the log­(θ/(1 – θ)) *versus* pH–p*K*_a_ graph near the point where pH equals p*K*_a_.^50^

#### Hemolysis Assay

Blood samples were collected from healthy donors providing informed consent under protocols approved by the Imperial College Healthcare Tissue Bank and the Local Research Ethics Committee in accordance with the Human Tissue Act 2004 under license MED_RS_11_014. The membrane-lytic activity of polymers was assessed at pH 7.4 (extracellular) and pH 5.5 (endosomal) conditions using a hemolysis assay in 0.1 M phosphate buffer (PB). Red blood cells (RBCs) were isolated from human blood samples following a standard procedure.^53^ Following centrifugation and three washes with 150 mM NaCl, RBCs were resuspended in 0.1 M PB at pH 7.4 or 5.5. Polymers at concentrations ranging from 1.5625 to 100 μM were incubated with RBC suspensions at both pHs in a sealed 96-well V-bottom plate for 1 h at 37 °C. After centrifugation at 1,500 rpm for 5 min, 100 μL of supernatant was transferred to clear 96-well plates for absorbance measurement at 541 nm using a Spectramax M5 plate reader (Molecular Devices, US). Percentage hemolysis was calculated by subtracting buffer-only negative controls and normalizing to 1% Triton X-100 positive controls. Error bars represent the standard deviation (SD) from three replicates.

#### *In Vitro* Transcription

saRNA encoding the replicase derived from the Venezuelan Equine Encephalitis Virus (VEEV) and firefly luciferase (fLuc) was synthesized using *in vitro* transcription (IVT) as previously described.^74^ Plasmid linearization was achieved by digesting DNA (2.5 μg) with MluI-HF enzyme for 2 h at 37 °C in a 20 μL reaction. Uncapped saRNA was synthesized with the HiScribe T7 High Yield RNA Synthesis Kit (New England Biolabs) and post-transcriptionally capped with the ScriptCap Cap 1 capping system (Cellscript) according to the manufacturer’s instructions. For fluorescent labeling, Cy5-UTP:UTP (1:4) or Cy3-UTP:Cy5-UTP:UTP (1:1:8) molar ratios were substituted during IVT reaction setup. Cy3/Cy5 mRNA used for TNP formulation assays was synthesized in the same manner following SpeI linearization of pGEM4Z-T7–5′UTR-NanoLuc-3′UTR-A64 (Addgene Plasmid #203350) but used without capping.

#### Ternary Nanoparticle (TNP) Formulation

The charge ratios of N/P and C/N in PP and TNP represent the molar ratio of amino groups (N) on pABOL to phosphate groups (P) on nucleic acids (either saRNA, mRNA, or pDNA) and carboxyl groups (C) on polyanions, respectively. TNP were formulated in two steps. Polyplexes were prepared by separately diluting pABOL to 4.5 mg/mL and saRNA to 0.1 mg/mL (mass ratio 45:1; N/P molar ratio of 37:1 for RNA) in equal volumes of Bis-Tris buffer (20 mM, pH 6.5), followed by vortexing for 10 s and incubation at room temperature for 15 min. Polybrene and jet-PEI polyplexes were formulated at an N/P = 10. Preformed PP and polyanions were combined in a 384-well plate (Aurora) at a 2:1 volume ratio and mixed by pipetting. The resulting TNP incubated at room temperature for 15 min prior to characterization.

The C/N ratio is calculated by first determining the net mass-to-charge ratio for each polymer component. For polyanions, the net mass/charge ratio equals the polyanion molecular weight divided by the number of carboxyl groups per polymer chain (determined from ^1^H NMR analysis of CEA incorporation). Similarly, for pABOL, the net mass/charge ratio equals the pABOL molecular weight divided by the number of amino groups per chain (calculated from the known polymer structure and GPC-determined molecular weight). The C/N ratio is then calculated as C/N = [Polyanion mass/(Net polyanion mass/charge)]: [pABOL mass/(Net pABOL mass/charge)].

#### PEGylated pABOL PP Preparation

PEGylated pABOL PP was prepared at the same N/P molar charge ratio as pABOL PP (37:1) following the above protocol. p­(PEG-*co*-ABOL) and saRNA were separately diluted in Bis-Tris buffer (20 mM, pH 6.5) at 11.5 and 0.1 mg/mL, respectively. Equal volumes were mixed and vortexed for 20 s to yield PEG–PP with a final saRNA concentration at 0.05 mg/mL.

### TNP Physical Characterization

#### Dynamic Light Scattering (DLS)

High throughput hydrodynamic diameter measurements were performed using a DynaPro Plate Reader III (Wyatt Instruments, USA). Samples (15 μL) were diluted 1:1 (v/v) in preparation buffer in a 384-well plate (Aurora) and measured in triplicate. For stability assessment, TNP solutions were mixed 1:1 (v/v) with buffers including 2× PBS (pH 7.4) with or without 2% FBS, or 0.2 M PB (pH 5.5 or 7.4) at 37 °C for 4 h before measurement. Wyatt data were presented as the intensity-weighted average of the cumulants fit using DYNAMICS software (version 7.9). For Figure 4, DLS measurements were performed on the same samples measured by SANS using a Zetasizer Nano (Malvern Panalytical, U.K.) following 20-fold dilution in 10 mM B-T buffer (pH 6.5), or 0.2 M PB (pH 7.4) prepared with 100% D_2_O. Malvern data were presented as the intensity-weighted Z-average of the cumulants fit using Zetasizer software (version 7.11).

#### Zeta Potential

Zeta potential measurements were performed using a Zetasizer. Samples were diluted in 1 mM NaCl, and three measurements were taken for each sample.

#### Transmission Electron Microscopy (TEM)

Freshly prepared samples (10 μL) were added directly to a CF400 mesh copper grid and allowed to adsorb for 60 s. The grid was then blotted with filter paper and stained by touching it to a drop of 2% (w/v) filtered uranyl acetate for 15 s. Excess stain was removed with filter paper, and the grid was air-dried overnight. Imaging was conducted using a TEM-2100 Plus electron microscope (JEOL, USA).

#### Gel Electrophoresis Assays of RNase and Heparin Resistance

To assay RNase resistance, naked saRNA, PP, and TNP samples were diluted 1:1 (v/v) in 2× PBS (pH 7.4) with 0.1 μg/mL RNase A (New England Biolabs) and incubated for 1 h at 37 °C or retained on ice without RNase A to serve as controls. RNase was degraded by further incubation with 2 mg/mL proteinase K (Ambion) for 15 min at 37 °C. RNA was purified using Chelex 100 cation exchange resin (BioRad), subjected to gel (0.5% v/v bleach, 1% w/v agarose, 1X SYBR gold) electrophoresis (1.75 h runtime at 110 V and 4 °C in 1× TAE buffer) along with an RNA Millenium Marker (Thermo Fisher), and visualized with a NuGenius XE gel imager (Syngene).

To assay heparin resistance, PP and TNP samples encapsulating fLuc saRNA were diluted 1:1 (v/v) in water with or without heparin (100, 200, 500 μg/mL), incubated for 1 h at 37 °C, and subjected to gel electrophoresis as above.

#### Förster Resonance Energy Transfer (FRET) Assay

A FRET assay was used to assess saRNA packaging within PP and TNP formulated using Cy3/Cy5 double-labeled saRNA. Samples were diluted 1:1 (v/v) in preparation buffer, 2× PBS (pH 7.4), 2× PBS + 10% FBS v/v, 0.2 M PB at pH 5.5, 0.2 M PB at pH 7.4, or 1 mg/mL heparin in water. Samples (30 μL, containing 0.25 μg saRNA) in a 384-well plate (Aurora) were incubated for 4 h at 37 °C. Fluorescence intensity was measured using a Spectramax M5 plate reader (Molecular Devices, USA) with excitation at 520 nm and emission wavelengths of 570 and 672 nm used for detecting Cy3 and Cy5, respectively. The FRET ratio was calculated as follows: FRET ratio = Intensity@672 nm/Intensity@570 nm.

#### Small Angle Neutron Scattering (SANS) Measurement

SANS measurements were conducted using samples formulated in Bis-Tris buffer (10 mM, pH 6.5) prepared with 100% D_2_O. The experiments were performed using the ZOOM SANS instrument at the ISIS Neutron and Muon Source, Rutherford Appleton Laboratory, Didcot, U.K. using a sample changer with a Julabo water bath for temperature control with a sample-to-detector distance of 4 m. All SANS data were collected using 1 mm path-length quartz cuvettes with a 150 μL sample volume at 25 °C. Samples were measured for 20 μA (SANS) proton current using a beamline configuration of L1 = L2 = 4 m collimation and sample–detector distances to give a scattering vector *q* = (4π/λ)­sin­(θ/2) range of 0.00416–0.77484 Å^–1^, where θ is the scattering angle and neutrons of wavelengths λ of 1.75–16.5 Å were used simultaneously by time-of-flight. Data reduction and background subtraction was performed using MantidPlot and scattering data was fitted with SASView v 5.0.5 using the DREAM data fitting algorithm.^75^ Samples were fitted using either a power-law model or Guinier-Porod model over defined *q* ranges. The core_shell_sphere model was applied to relevant samples to analyze the internal mesoscopic structure of formulations. For the core_shell_sphere model, the fitting parameters included core radius, shell thickness, SLD of both the core and shell, and the polydispersity of the shell thickness. Due to the high sample polydispersity, the PDI of both core and shell were fixed at 0.3. The core radius and shell thickness were first fixed at expected values. Core SLD (comprising polymers and saRNA) and PEG-containing shell SLD were constrained between 1 and 6.3 × 10^–6^ Å^–2^, based on polymer SLD of around 1 × 10^–6^ Å^–2^ and 100% D_2_O buffer SLD of 6.3 × 10^–6^ Å^–2^. Information including all fitted parameters, fitting *q* ranges and errors in the fits are included in Tables S3 and S4.

#### Nano Flow Cytometry (nanoFCM)

Polyplexes were formulated with Cy5-saRNA and further mixed with AF488-P5 at C/N = 1 to form dual-labeled TNPs. Nanoparticles (PP and TCN) diluted 1:1000 in formulation buffer were analyzed using a Flow NanoAnalyzer N30 instrument (NanoFCM Co., Ltd., U.K.) and compared to buffer only. Greater than 10,000 events were recorded on two channels (488/10 and 670/30) adjusted to 10/50 mW and 20/100 mW, respectively, following alignment with silica QC beads. Size calibration curves were generated using the S17M-MV size standard (150–1000 nm) (NanoFCM Co., Ltd.). Data were plotted as Cy5-saRNA vs AF488-P5 using FlowJo software (version 10.4, BD Biosciences, USA). to reveal Cy5+/AF488+ loaded TNP, Cy5+/AF488- loaded PP, and Cy5-/AF488+ empty polyelectrolyte particles.

#### Cell Culture

The mouse dendritic cell line DC2.4 was cultured in complete RPMI 1640 medium, supplemented with 4.5 g/L glucose, 10% FBS, 100 U/mL penicillin-streptomycin, 10 mM HEPES, 10 mM MEM, and 0.05 mM β-mercaptoethanol (BME). The cells were maintained in a humidified incubator at 37 °C with 5% CO_2_. DC2.4 cells were regularly tested for mycoplasma contamination using a DNA-based PCR test and were confirmed to be negative.

#### *In Vitro* Cell Internalization Assay

DC2.4 cells were seeded in a 24-well plate at a density of 2 × 10^5^ cells per well in 500 μL complete RPMI 1640 medium and cultured overnight at 37 °C in 5% CO_2_. Prior to treatment, medium was replaced with either OptiMEM or fresh complete RPMI 1640 medium without BME. Cells were then treated with lipofectamine mMAX (prepared according to manufacturer’s instructions), PP or TNP formulations containing Cy5-saRNA at a dose of 500 ng per well for 4 h. Following treatment, cells were washed with PBS, detached with 0.05% trypsin, and collected by centrifugation at 1,200 rpm for 5 min. The cell pellet was washed with PBS and fixed with 4% paraformaldehyde (PFA) for 15 min at room temperature. The fixed cells were then washed and resuspended in 300 μL PBS containing 1% BSA. Flow cytometric analysis was performed using a LSRFortessa flow cytometer (BD Biosciences. USA), with gating based on forward scatter and side scatter. A total of 10,000 events were collected for each treatment condition with three biological replicates. The percentage of Cy5-positive cells and the mean fluorescence intensities (MFI) were calculated using FlowJo software.

#### *In Vitro* Transfection Assay

DC2.4 cells were seeded in a 96-well plate at a density of 5 × 10^4^ cells per well in 100 μL complete RPMI 1640 medium and cultured overnight at 37 °C in 5% CO_2_. Prior to treatment, medium was replaced with either OptiMEM or fresh complete RPMI 1640 medium. Cells were then treated with mMAX, PP or TNP formulations containing fLuc saRNA at a dose of 200 ng per well for 4 h. Following treatment, the medium was removed and replaced with 100 μL of complete RPMI 1640 medium, and cells were cultured for an additional 20 h. Luciferase expression was measured 24 h post-transfection by removing 50 μL medium and replacing with 50 μL ONE-Glo reagent, which was mixed thoroughly by pipetting. The total volume was transferred to a white 96-well plate (Costar, U.K.) and luminescence was measured using an EnVision multimode microplate reader (PerkinElmer, USA).

#### *In Vitro* Cytotoxicity Assay

DC2.4 cells were treated with PP and TNP prepared with fLuc saRNA at a dose of 200 ng per well following the protocol described for the transfection assay. At 24 h post-treatment, 10 μL CCK-8 reagent was added to each well, and the cells were incubated for an additional 2 h. Absorbance was measured at 450 nm using a Spectramax M5 plate reader (Molecular Devices, USA). Background readings from wells containing only CCK-8 solution and medium were subtracted from all measurements. Absorbance values were then normalized to those obtained from untreated cells to calculate cell viability.

#### *In Vitro* Intracellular Trafficking Assay

DC2.4 cells were seeded in an 8-well μ-Slide (ibidi, Germany) at a density of 4 × 10^4^ cells per well in 300 μL complete RPMI 1640 medium and cultured overnight at 37 °C in 5% CO_2_. Prior to treatment, medium was replaced with OptiMEM or fresh complete RPMI 1640 medium. Cells were then treated with PP or TNP formulated with Cy5-saRNA, FITC-labeled pABOL, and AZDye405-labeled polyanions at a dose of 200 ng per well for a 1 h. Lysotracker Red DND-99 was added 30 min prior to the end of the incubation at a final concentration of 75 nM to stain the lysosomal compartment. Following treatment, cells were washed with PBS and fixed with methanol-free 4% PFA for 15 min at room temperature, and washed again with PBS before imaging. Imaging was performed using a Zeiss LSM780 confocal microscope (Germany) with a 63× objective lens. Image analysis was conducted using FIJI software with consistent brightness/contrast adjustments applied uniformly across all experimental groups to ensure valid comparisons. Colocalization quantification was quantified using Manders’ overlap coefficients. An additional time point at 4 h was also assessed and imaged under the same conditions.

### Molecular Dynamics (MD) Simulation

#### Parameterization of Polymers

The parametrization scheme for the bonded parameters in the coarse-grained (CG) models of PEG as reported elsewhere.^76^ and those of polymethyl acrylate was adapted from ref 77. A minimal parametrization scheme was applied to saRNA. For pABOL and polyanions, a bottom-up approach was employed (adapted from ref 68), using reference all-atom (AA) models. Each polymer structure was mapped to corresponding CG beads, following a ∼4 heavy atoms:1 CG bead, consistent with the standard MARTINI framework. Specific details regarding CG mapping and the assignment of MARTINI bead types are provided in Figure S22.

We generated an AA model of each polymer segment and performed AA MD simulations in water, with neutralizing Na+ or Cl– counterions. From these simulations, we extracted “virtual” CG trajectories, where the center of mass of each group of atoms corresponding to a CG bead defines the coordinate of “virtual” CG bead. Using these bead coordinates, we computed the distributions of bonds and angles from the virtual CG trajectories, which were approximately Gaussian. These distributions were then used to derive CG bond and angle parameters. Specifically, the equilibrium bond lengths and angles were set as the average values from the distribution, and the harmonic force constants were determined based on the variance (σ^2^) of the equilibrium values over the entire trajectory $\left(k=\frac{k_{B}T}{\sigma^{2}}\right)$ . With these bond and angle parameters, we performed CG MD simulations and compared the resulting bond and angle distributions to those obtained from the virtual CG trajectories. The comparisons, as shown in Figures S23 and S24, demonstrate good agreement between the two methods, validating the parametrization approach.

#### All-Atom (AA) Polymer Systems

The simulation protocol is adapted from ref 78. Details are included in Supporting Information. The general Amber force field (GAFF) with SPC/E water model was applied,^79^ and all molecules were assigned partial charges from restrained electrostatic potential (RESP) charges^80^ using Gaussian 03 Revision D.01.^81^ AA simulations was conducted using Amber18.^82^ The temperature for all simulations was controlled using a Langevin thermostat with a collision frequency of 2 ps^–1^.^83^ Bond lengths involving hydrogen were constrained using the SHAKE algorithm.^84^ When pressure was specified, an isotropic Berendsen barostat was used with a time constant 1.0 ps.^85^ The simulation time step was set to 2 fs with MD leapfrog integrator. Electrostatic interactions were calculated using Particle Mesh Ewald (PME) method with a real-space cutoff of 8.0 Å.^86^ The van der Waals interaction cutoff was also set to 8.0 Å. After a short minimization using steepest descent method (switching to conjugate gradient descent method after 100 cycles), the system was heated from 0 to 300 K in 18 ps and held for 2 ps with constant volume, then was equilibrated at 300 K for 2 ns with a specified pressure of 1 bar. The production simulation was 40 ns under an NVT ensemble. Periodic boundary conditions were used in all directions.

#### Coarse-Grained (CG) Polymer Systems

MARTINI force field^87^ version 2.3P was applied, and all simulations were performed with GROMACS 5.1.5 ^88^ with a leapfrog integrator. The simulation protocol was adapted from ref 68. The temperature was controlled using a velocity-rescale thermostat with a time constant 1.0 ps.^89^ Isotropic pressure coupling was controlled with a compressibility of 3 × 10^–4^ bar^–1^ using the Berendsen barostat with a time constant of 5.0 ps during equilibration runs, and Parrinello–Rahman barostat with a time constant of 12.0 ps during production runs.^90^ Neighbor lists were constructed every 20 steps using a Verlet scheme with a cutoff of 1.1 nm. van der Waals interaction was modeled with cutoff scheme with the potential shifted to zero at 1.1 nm. Electrostatic interactions were calculated with a dielectric constant of 2.5 and the PME method with a short-range cutoff of 1.1 nm, a Fourier spacing of 0.12 nm, and fourth-order interpolation. The total energy of the system was minimized for 5000 steps or until the maximum force converges to 1000 kJ/mol·nm. The temperature was maintained at 300 K, and the pressure was set to 1 bar. Initial equilibration was performed for 5 ns with a time step of 10 fs, followed by 15 ns with a time step of 15 fs, and finally 25 ns with a time step of 20 fs. Production runs were performed for 50 ns with a time step of 20 fs.

For the system setup, 50 14mer pABOL chains and two 50mer RNA chains were assembled into a spherical box with a radius of 50 Å, followed by 35 polyanion chains assembling into a spherical box with a radius of 80 Å, forming a pseudo core–shell structure using Packmol.^69^ The system was then solvated with 200,000 water beads and corresponding ions. Detailed system specifications are shown in Tables S5 and S6. All the visualization of simulation trajectories was performed using Visual Molecular Dynamics (VMD).^91^

#### Statistical Analysis

Statistical analysis was performed using Prism 10.0 (GraphPad). The specific statistical methods were indicated in the figure legend. Statistical significance is denoted as follows: ****p < 0.0001, ***p < 0.001, **p < 0.01, *p < 0.05, ns means not significant.

## Supplementary Material



## Notes

Detailed synthetic schemes and GPC, NMR, and SANS characterization data as well as additional TNP formulation characterization, *in vitro* microscopy, and molecular dynamics model development mentioned in the text (PDF)
