# Supplementary material for: Polyanion Chemistry Engineers Ternary RNA Nanoparticle Structure/Function from the Inside-Out
Source: ACS Nano. 2026 Jan 27;20(5):4508–26. doi: 10.1021/acsnano.5c19683 (PMC12895518; doi:10.1021/acsnano.5c19683)
Supplement: Supplementary file 1 [file nn5c19683_si_001.pdf]

Supporting Information

Polyanion chemistry engineers ternary RNA nanoparticle structure/function from the inside-out

Lijun Hu[1,2,†], David J. Peeler[1,2,3,†], Tianyi Jin[4], James J. Doutch[5], Baihao Shao[1,2], Jonathan Yeow[1], Li Ma[1,2], Hanna M.G. Barriga[6], Jiaqing Tang[1,2], Xuan Cao[7], Chenchen Liu[1,2], Christopher L. Grigsby[6], Alfredo Alexander-Katz[8], Robin J. Shattock[3], and Molly M. Stevens[1,2,*]

1 Kavli Institute for Nanoscience Discovery, Department of Physiology, Anatomy and Genetics, Department of Engineering Science, University of Oxford, Oxford OX1 3QU, United Kingdom

2 Department of Materials, Department of Bioengineering, Institute of Biomedical Engineering, Imperial College London, London SW7 2AZ, United Kingdom

3 Department of Infectious Disease, Faculty of Medicine, Imperial College London, SW7 2AZ, United Kingdom

4 Department of Chemical Engineering, Massachusetts Institute of Technology, Cambridge, Massachusetts 02139, United States of America

5 ISIS Neutron and Muon Source, Harwell Campus, Oxford OX1 10QX, UK

6 Department of Medical Biochemistry and Biophysics, Karolinska Institute, Huddinge, Stockholm 171 77, Sweden

7 Department of Earth Sciences, University of Oxford, Oxford OX1 3AN, United Kingdom; Department of Earth Sciences, University of Cambridge, Cambridge CB2 3EQ, United Kingdom

8 Department of Materials Science and Engineering, Massachusetts Institute of Technology, Cambridge, Massachusetts 02139, United States of America

[†]These authors contributed equally to this work.

*Corresponding author: Molly M. Stevens molly.stevens@dpag.ox.ac.uk

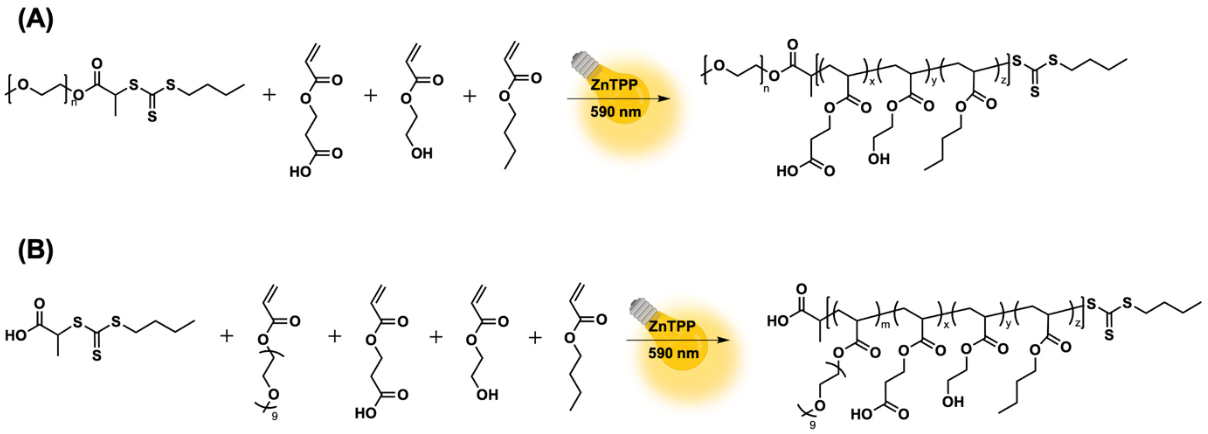

Scheme S1. Scheme depicting ZnTPP-initiated PET-RAFT polymerization from macro-CTA, mPEG-BTPA (A) and BTPA (B) under yellow light in 96-well plates. Light bulb adapted from BioRender.com.

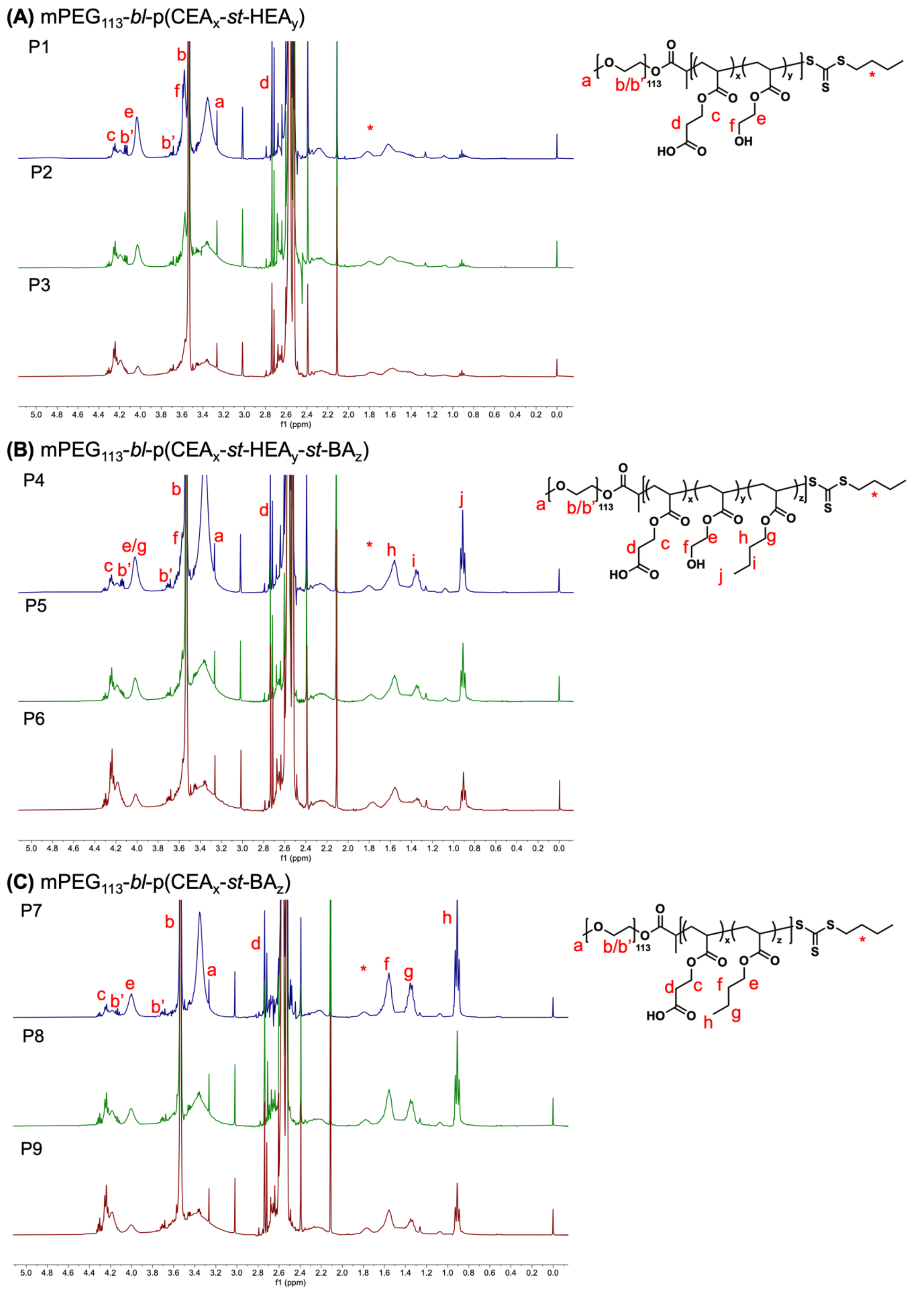

Figure S1. ^{1}H NMR spectra of (A) P1-P3, (B) P4-P6 and (C) P7-P9 in DMSO-*d*$_6$. Note: b' denotes the terminal PEG unit, asterisk (*) denotes the peaks corresponding to the CTA.

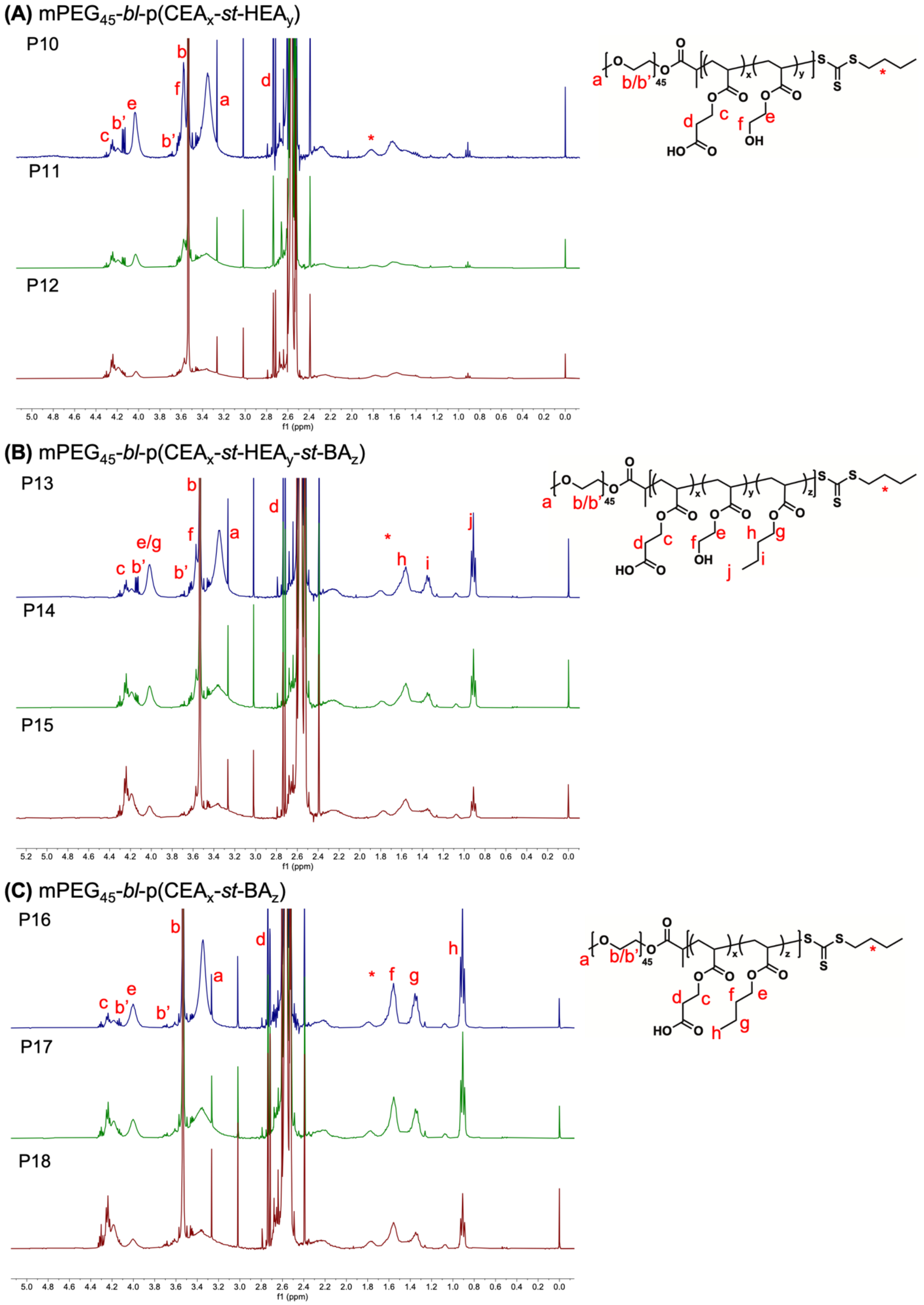

Figure S2. **^{1}H NMR spectra of (A) P10-P12, (B) P13-P15, and (C) P16-P18 in DMSO-*d*₆. Note: b' denotes the terminal PEG unit, asterisk (*) denotes the peaks corresponding to the CTA.**

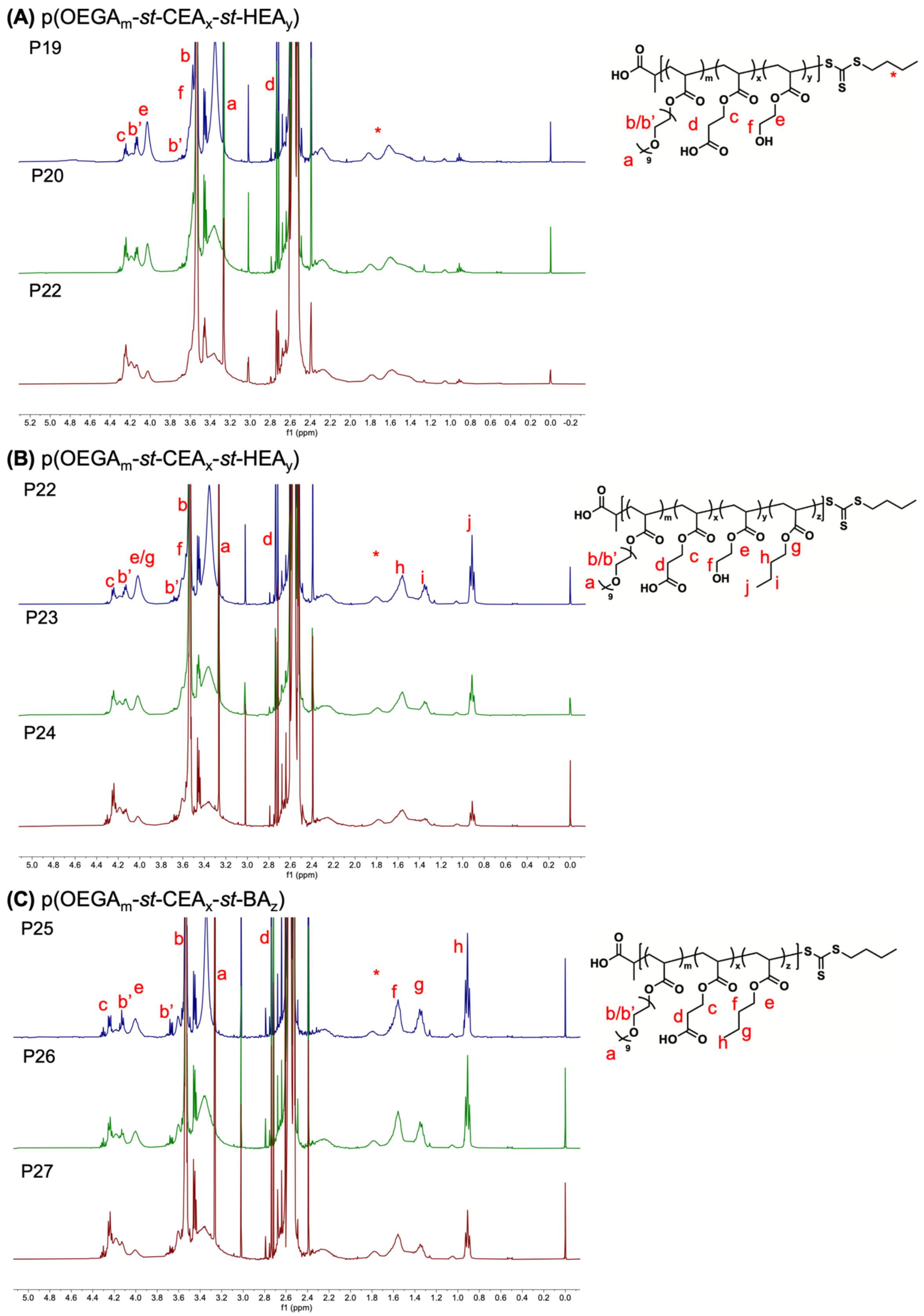

Figure S3. **^{1}H NMR spectra of (A) P19-P21, (B) P22-P24, and (C) P25-P27 in DMSO-*d*$_6$. Note: b' denotes the terminal PEG unit, asterisk (*) denotes the peaks corresponding to the CTA.**

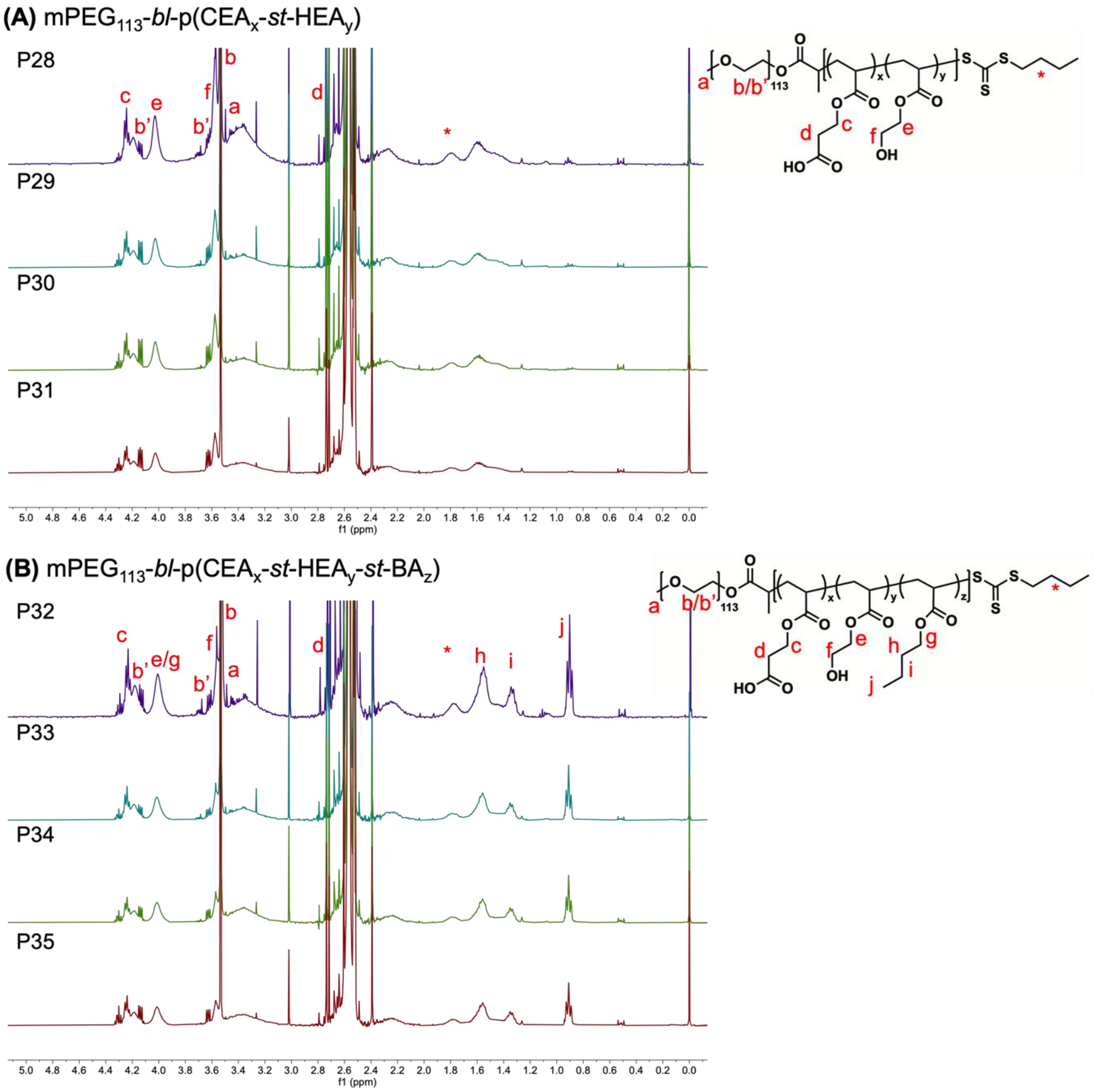

Figure S4. **^{1}H NMR spectra of (A) P28-P31, and (B) P32-P35 in DMSO-*d₆*. Note: b' denotes the terminal PEG unit, asterisk (*) denotes the peaks corresponding to the CTA.**

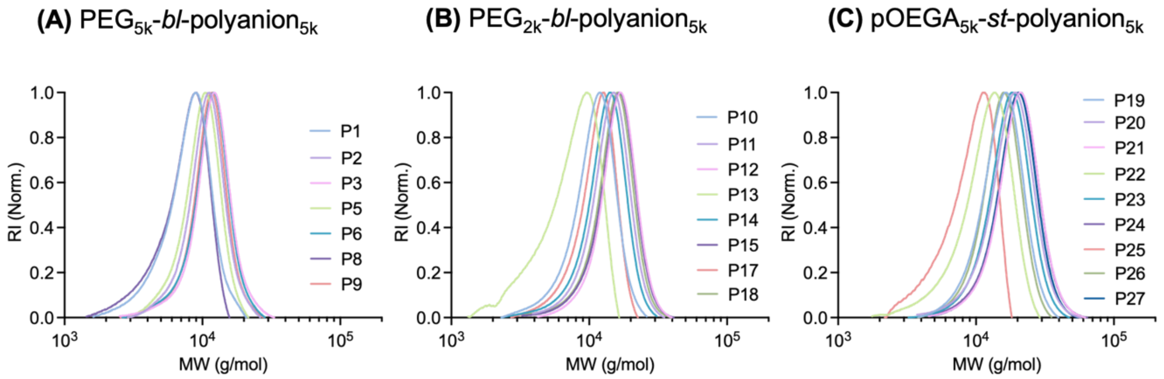

Figure S5. Aqueous GPC molecular weight distributions for (A) P1-P9, (B) P10-P18, and (C) P19-P27. Signal was not detected for P4, P7, P16.

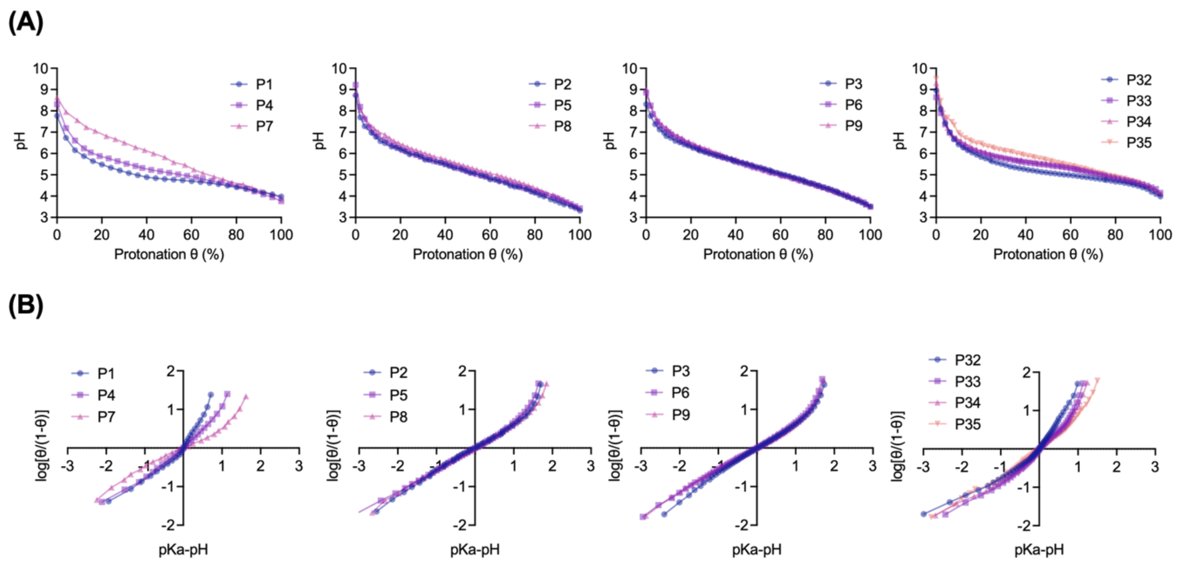

Figure S6. Polyanion titration. (A) pH titration curves for PEG$_{5k}$-*bl*-polyanions with normalization of titration coordinate to a measure of the degree of protonation θ. (B) Hill plots based on a cooperativity modification of the Henderson−Hasselbach equation.

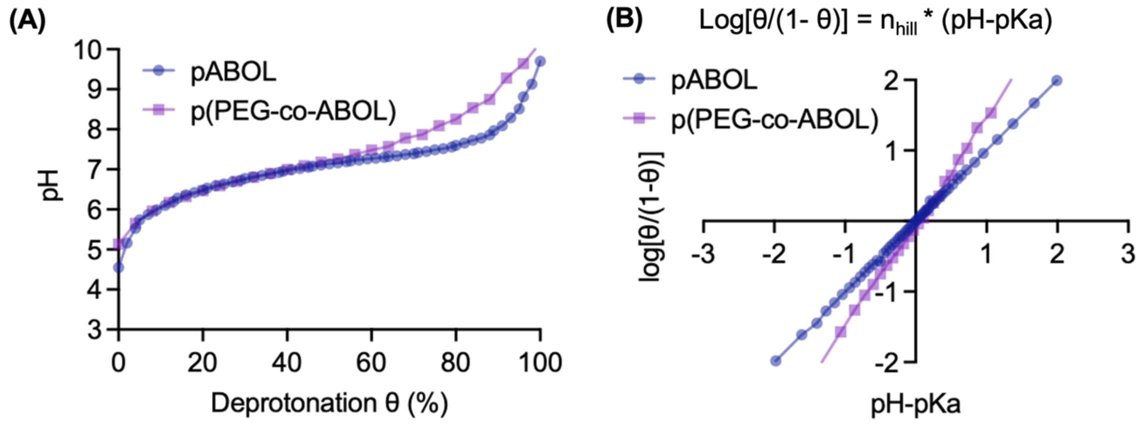

Figure S7. Polycation titration. (A) pH titration curves for pABOL and p(PEG-*co*-ABOL) with normalization of titration coordinate to a measure of the degree of deprotonation θ. (B) Hill plots based on a cooperativity modification of the Henderson−Hasselbach equation.

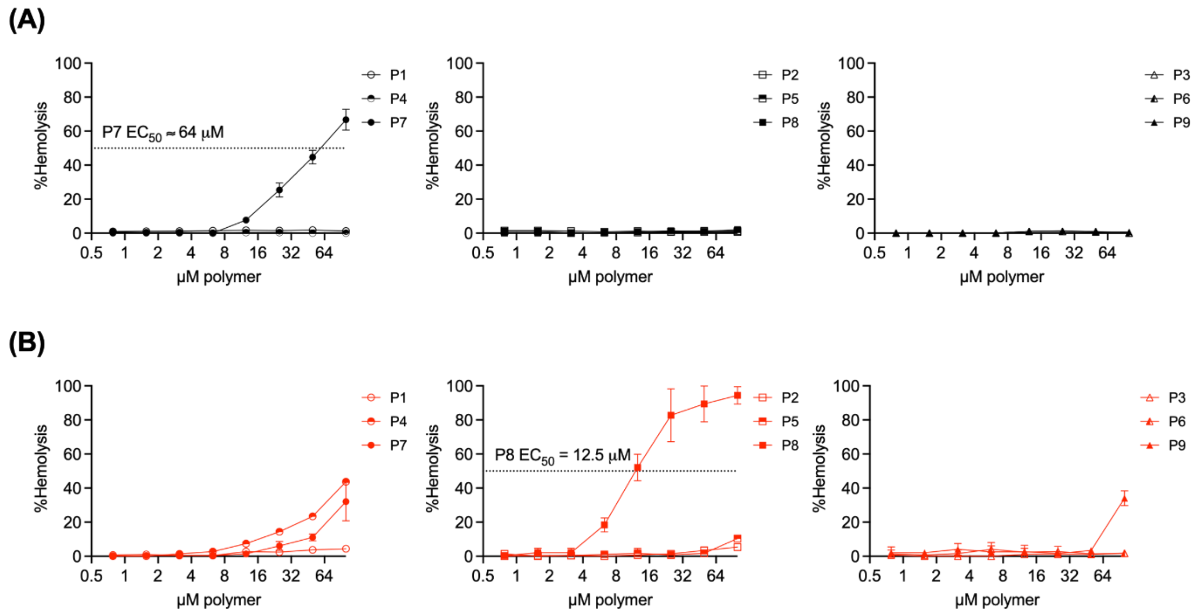

Figure S8. PEG$_{5k}$-*bl*-polyanion$_{5k}$ hemolysis. Polyanions were incubated with human erythrocytes at series of concentrations at for 1 h in (A) PB pH 5.5 and (B) PB pH 7.4. Data shown as mean ± SD, N = 3.

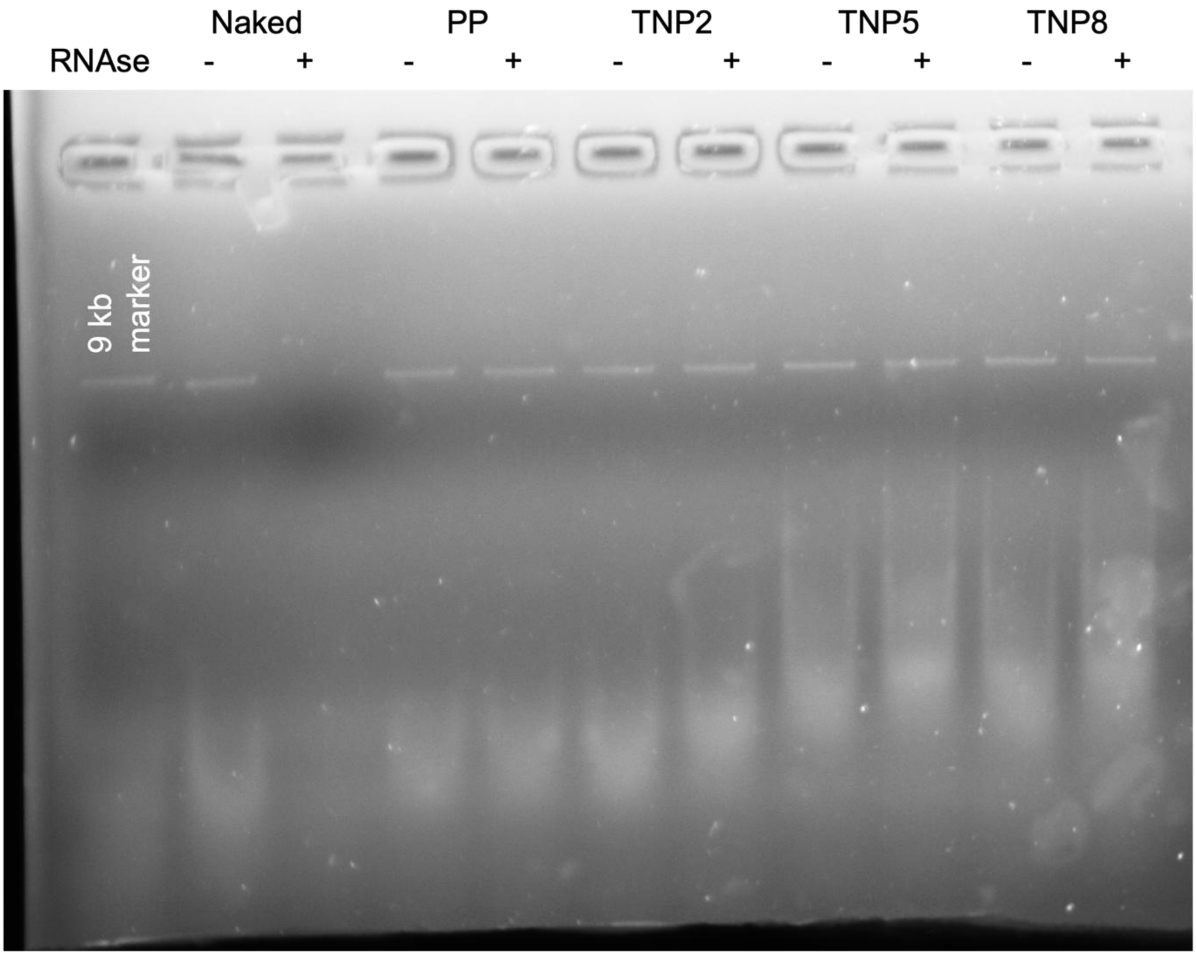

Figure S9. RNAse resistance. PP or C/N =1 TNP2, 5, or 8 encapsulating 250 ng fLuc saRNA were incubated either on ice or in PBS + 0.1 µg/mL RNAse A for 1 h at 37°C, incubated with 2 mg/mL proteinase K to digest RNAse for 15 min at 50°C, purified saRNA using Chelex 100 cation exchange resin, and visualized after gel electrophoresis in a 1% agarose gel in TAE buffer (1.75 h,110 V, 4 °C) along with the RNA Millenium Marker.

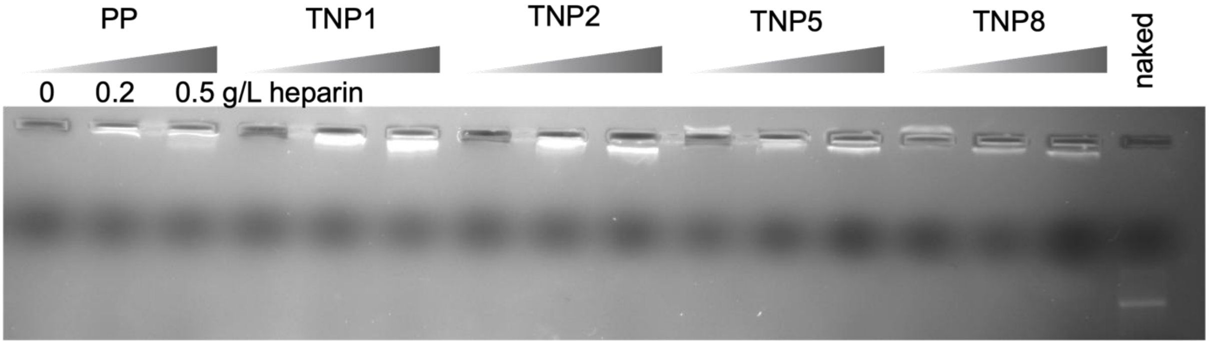

Figure S10. Heparin resistance. PP or C/N =1 TNP1, 2, 5, or 8 encapsulating 500 ng saRNA were incubated in 0.2 or 0.5 g/L heparin for 1 h at 37°C and subjected to gel electrophoresis to visualize unpackaging.

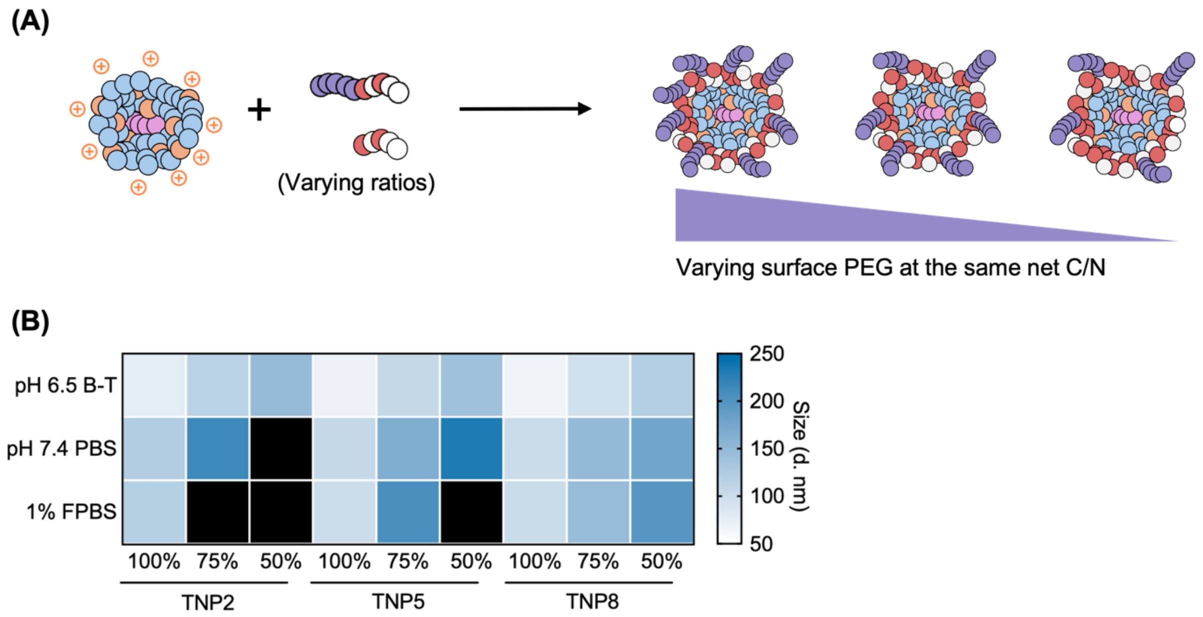

Figure S11. PEG density and polyanion hydrophobicity govern TNP colloidal stability. (A) Illustration scheme of TNP formulated with different ratios of PEGylated to non-PEGylated polyanions with C/N fixed at 1. (B) DLS measurement of intensity-weighted mean hydrodynamic diameter stability in salt and protein. Data shown as mean of three replicates, N = 3.

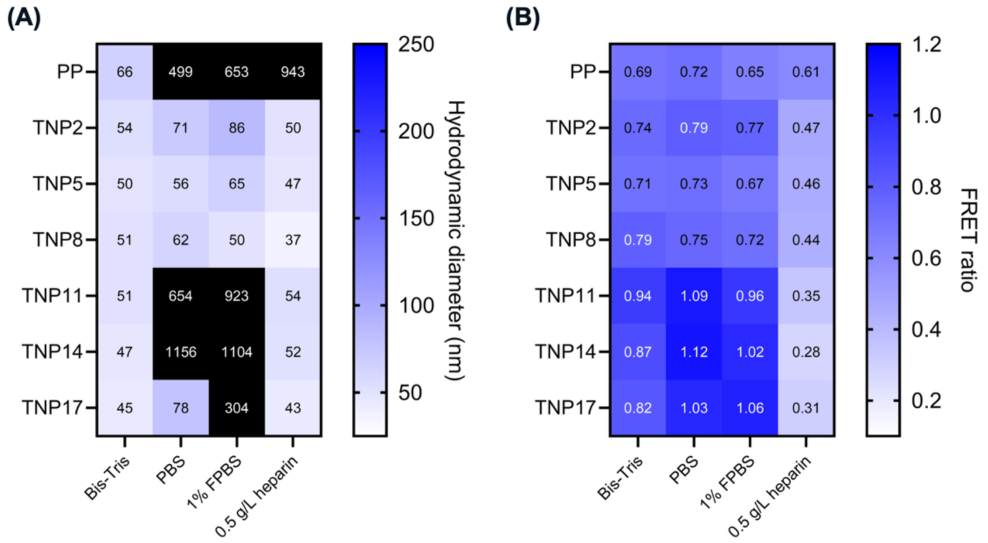

Figure S12. Formulation and stability screening of pABOL-mRNA TNP. pABOL-Cy3/Cy5-mRNA TNP formulated at C/N = 1 were added to various buffers and characterized by (A) DLS measurement of intensity-weighted mean hydrodynamic diameter and, following incubation at 37 °C for 4 hours, by (B) plate reader in terms of Cy3/Cy5 FRET efficiency indicating tightness of mRNA packaging. Heatmap in black indicates exceeding the upper range. (N=1; n=3)

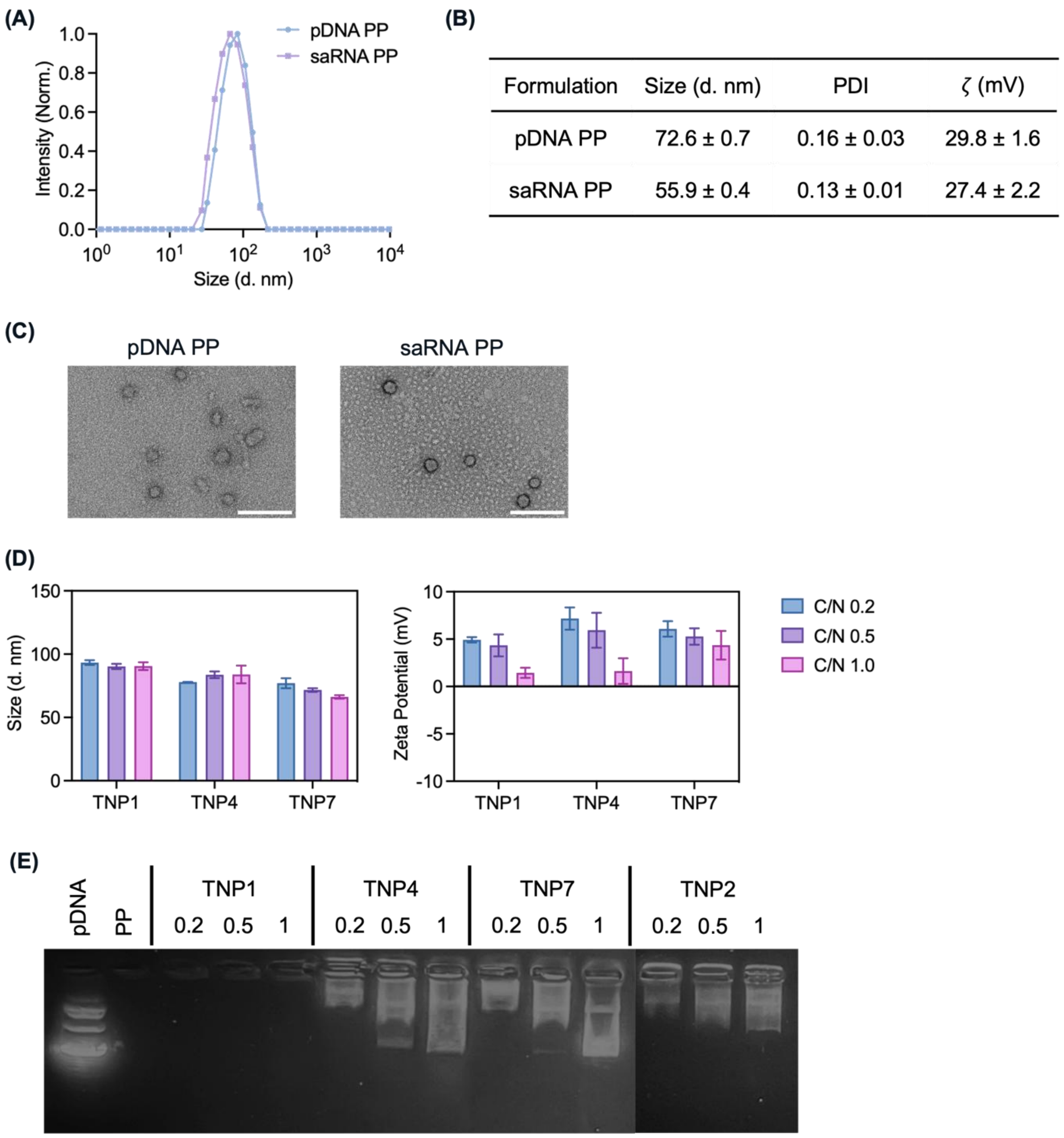

Figure S13. Characterization of pABOL/pDNA TNP. (A) Particle size distribution by intensity. (B) Size stability in terms of intensity-weighted mean hydrodynamic diameter, PDI and zeta potential (ζ). Data shown as mean ± SD, N = 3. (C) TEM images. Scale bar = 200 nm. (D) Hydrodynamic diameter (intensity average) and zeta potential of pDNA TNP formulated at different C/N ratios. Data shown as mean ± SD, N = 3. (E) Agarose gel electrophoresis of naked pDNA, pDNA PP, and pDNA TNP formulated with different polyanions at various C/N ratios. Numbers represent C/N ratios. Charge density: P2 (50%) >P1 = P4 = P7 (25%); Hydrophobicity: P7>P4>P1. (Data for TNP2 were obtained from an independently run gel)

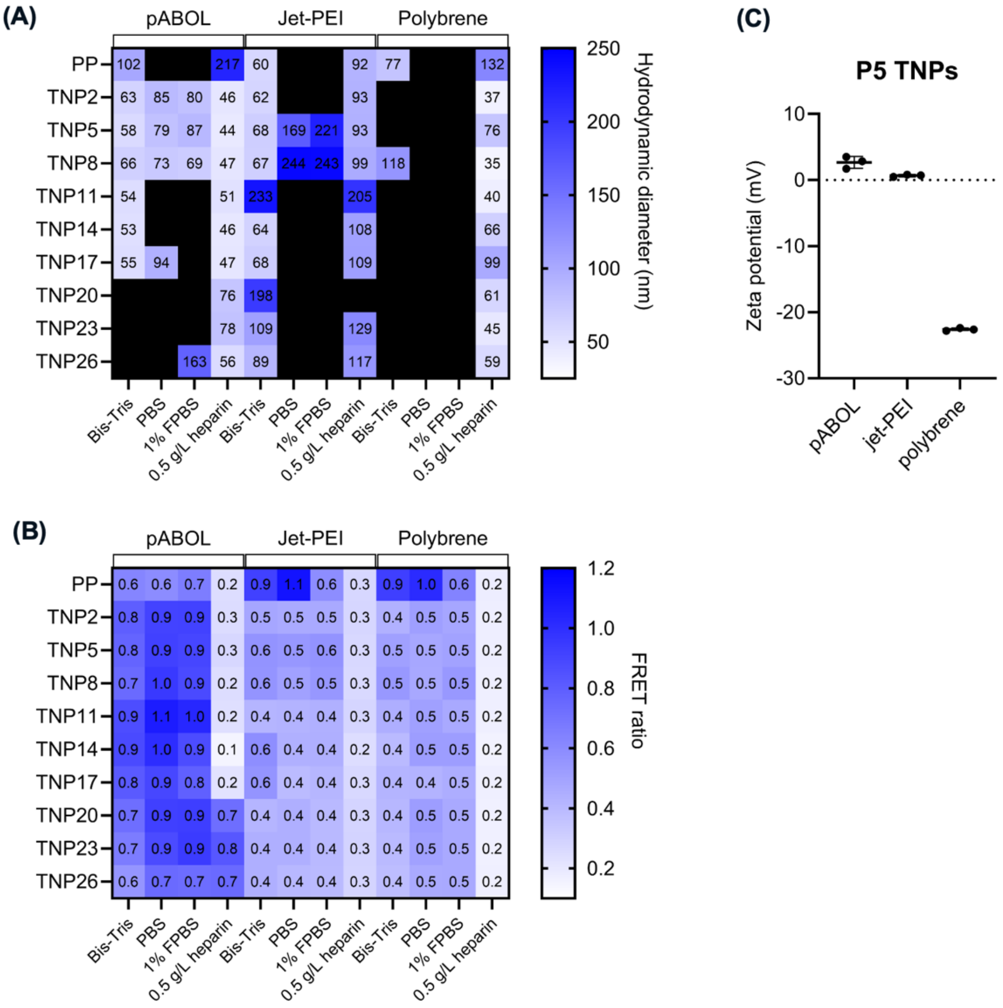

Figure S14. Formulation and stability screening of saRNA TNP with alternative polycations. Polyplexes were formulated with Cy3/Cy5-saRNA and either pABOL (N/P = 37), Jet PEI (N/P = 10), or polybrene (N/P = 10) before further mixing with various coating polymers to form TNP at C/N = 1. Nanoparticles were added to various buffers and characterized by (A) DLS in terms of intensity-weighted mean hydrodynamic diameter and, following incubation at 37 °C for 4 hours, by (B) plate reader in terms of Cy3/Cy5 FRET efficiency indicating tightness of mRNA packaging. (C) Zeta potential measurement of pABOL-, JetPEI-, and polybrene-TNP5 to confirm TNP assembly. Heatmap in black indicates exceeding the upper range. (N=1; n=3)

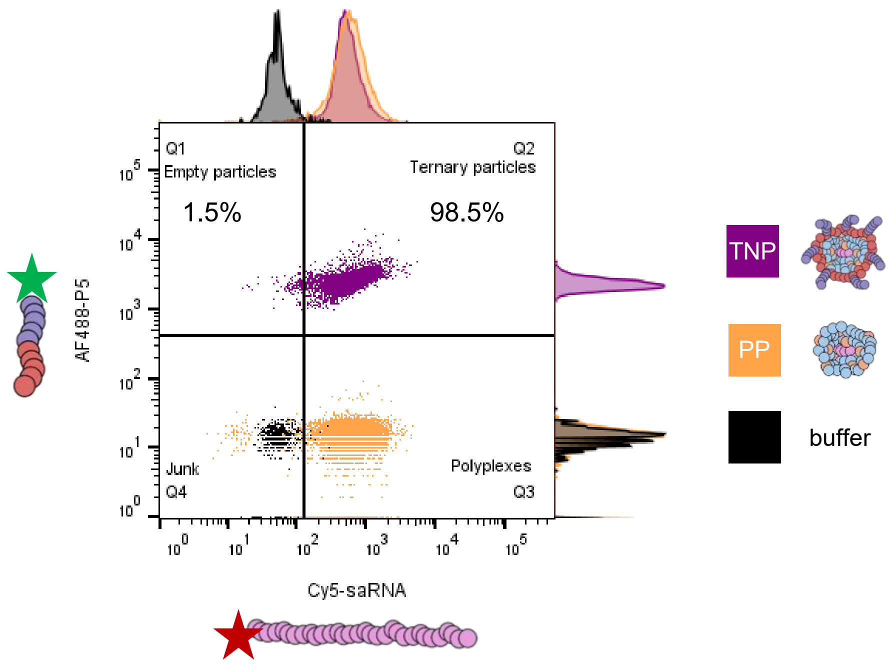

Figure S15. Nano flow cytometry characterization of saRNA PP and TNP. Polyplexes were formulated with Cy5-saRNA and further mixed with AF488-P5 at C/N = 1 to form dual-labeled TNP. Nanoparticles (n $\geq$ 10,000 particles) or blank buffer were analyzed by nanoFCM and plotted as Cy5-saRNA *vs.* AF488-P5 to reveal saRNA-loaded TNP (Cy5$^+$/AF488$^+$), saRNA-loaded PP (Cy5$^+$/AF488$^-$), and empty polyion complexes (Cy5$^-$/AF488$^+$). Overlaid percentages are for the TNP sample using the gating strategy shown in the plot.

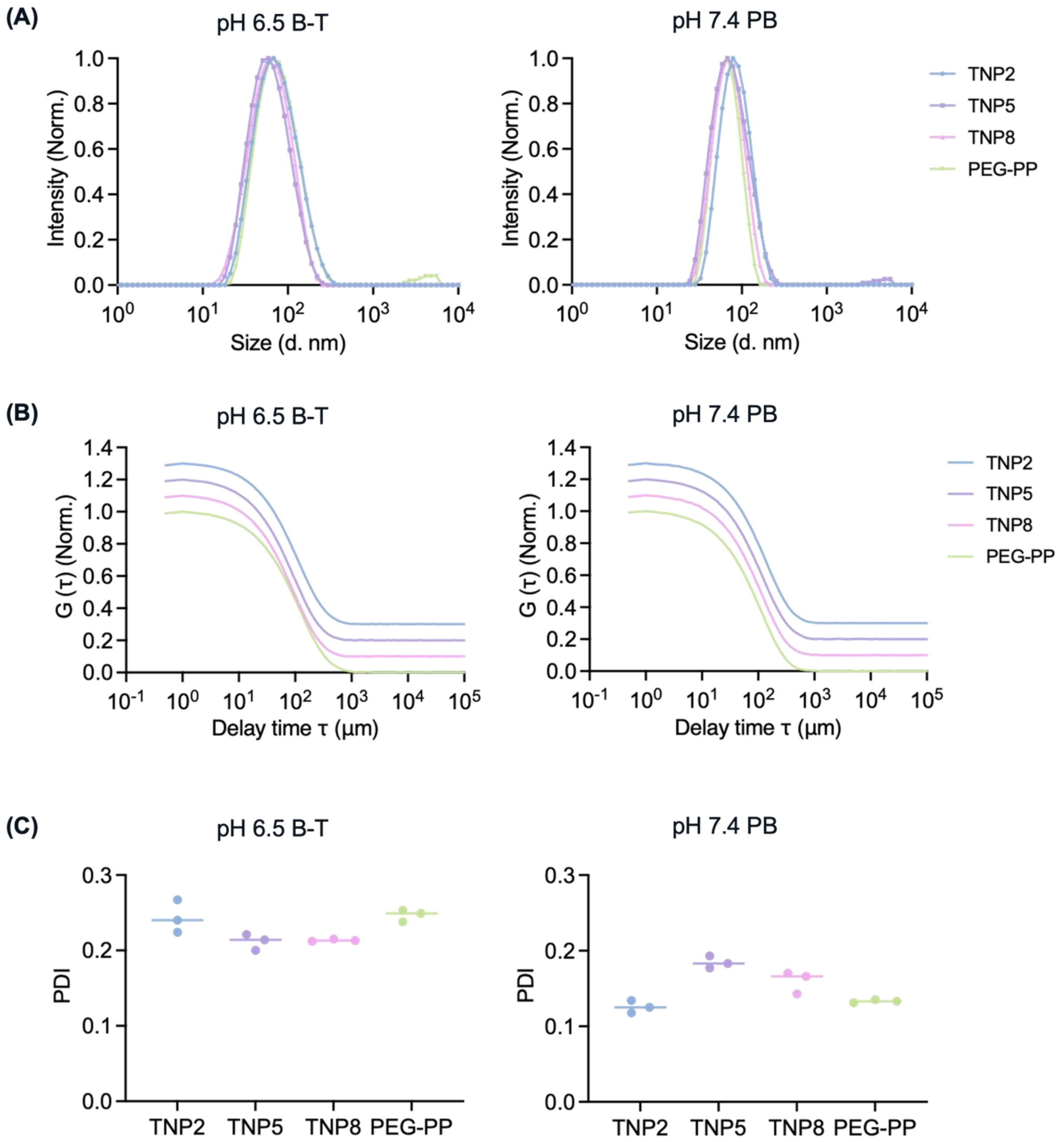

Figure S16. DLS measurements of (A) hydrodynamic diameter by intensity distribution, (B) autocorrelation function curves (offset for clarity), and (C) polydispersity index (PDI for TNP2, TNP5, TNP8, and PEG-PP formulations. Data shown as mean ± SD, N = 3.

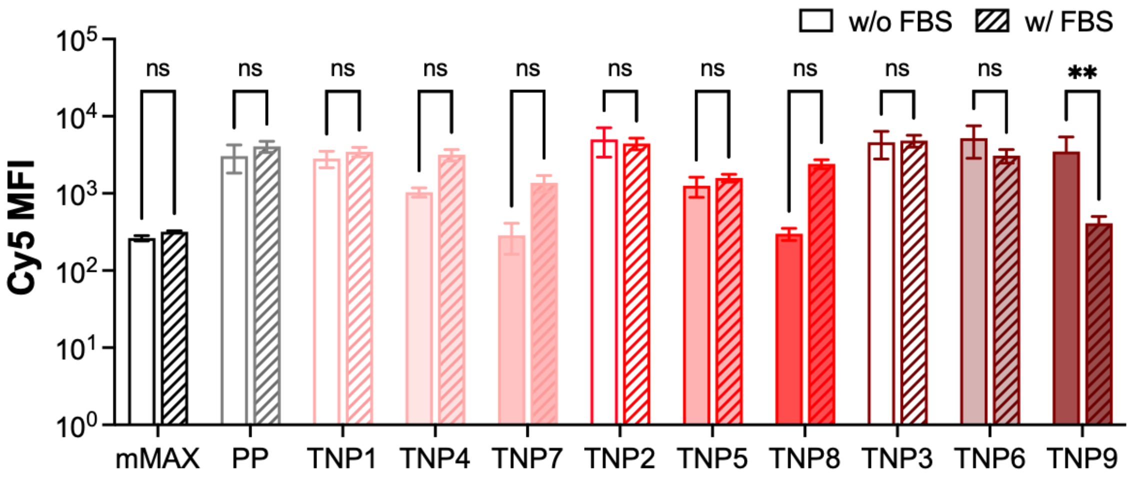

Figure S17. Mean fluorescence intensity (MFI) of Cy5-saRNA uptake in singlet DC2.4 cell events analyzed by flow cytometry after 4 hours of incubation with various PP and TNP formulations. Data shown as mean ± SD, N = 3. Statistical significance was calculated using one-way analysis of variance (ANOVA) and Tukey's multiple comparisons. **p < 0.01, ns means not significant.

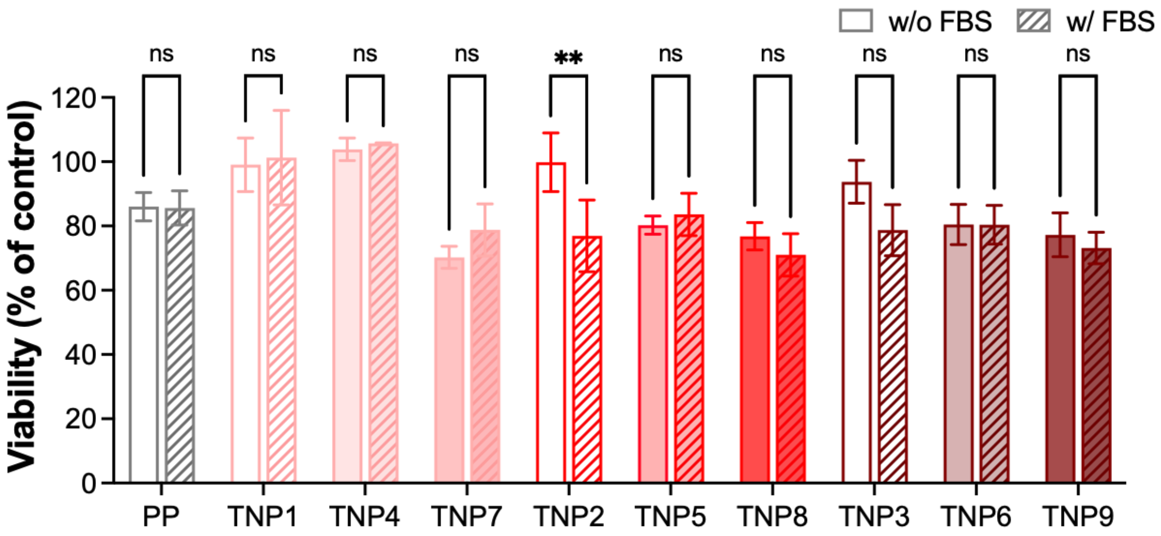

Figure S18. Impact of saRNA formulations on cell metabolism in DC2.4 cells assessed by the CCK-8 assay. Cell viability was determined by normalizing to the relative metabolic activity of untreated cells. Data shown as mean ± SD, N = 3. Statistical significance was calculated using one-way analysis of variance (ANOVA) and Tukey's multiple comparisons. **p < 0.01, ns means not significant.

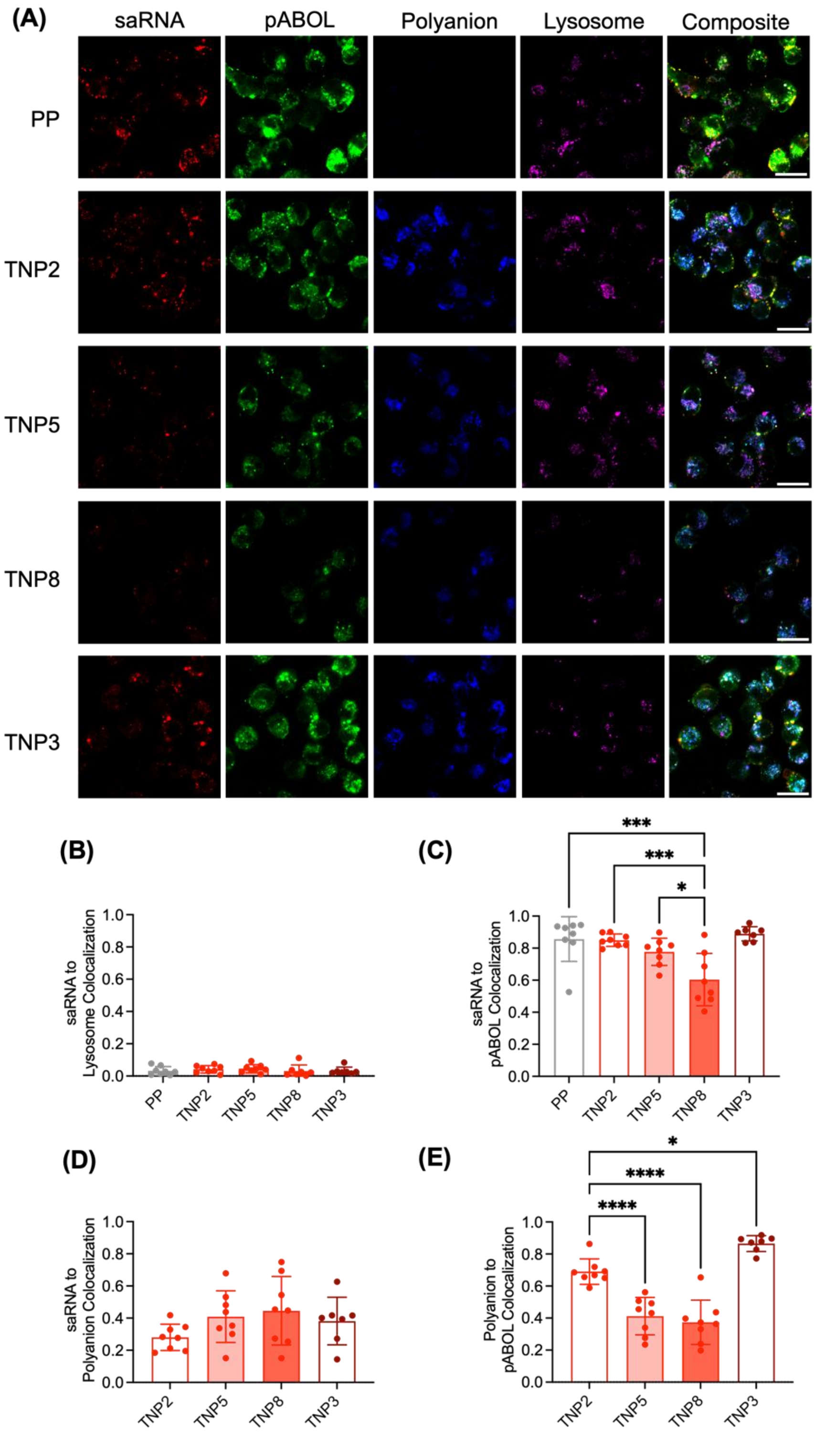

Figure S19. (A) Representative confocal microscopy images of DC2.4 cells treated with PP or TNP containing Cy5-labeled saRNA, FITC-pABOL, and AZDye405-polyanions for 1 h in the absence of FBS. The acidic compartments were stained with Lysotracker Red. Scale bar = 20 μm. Manders' overlap coefficients of colocalization of (B) Cy5-saRNA and Lysotracker, (C) Cy5-saRNA and FITC-pABOL, (D) Cy5-saRNA and AZDye405-polyanion, (E) AZDye405-polyanion and FITC-pABOL. Data shown as mean ± SD, N > 7. Statistical significance was calculated using one-way analysis of variance (ANOVA) and Tukey's multiple comparisons. ****$p < 0.0001$, ***$p < 0.001$, *$p < 0.05$, ns means not significant.

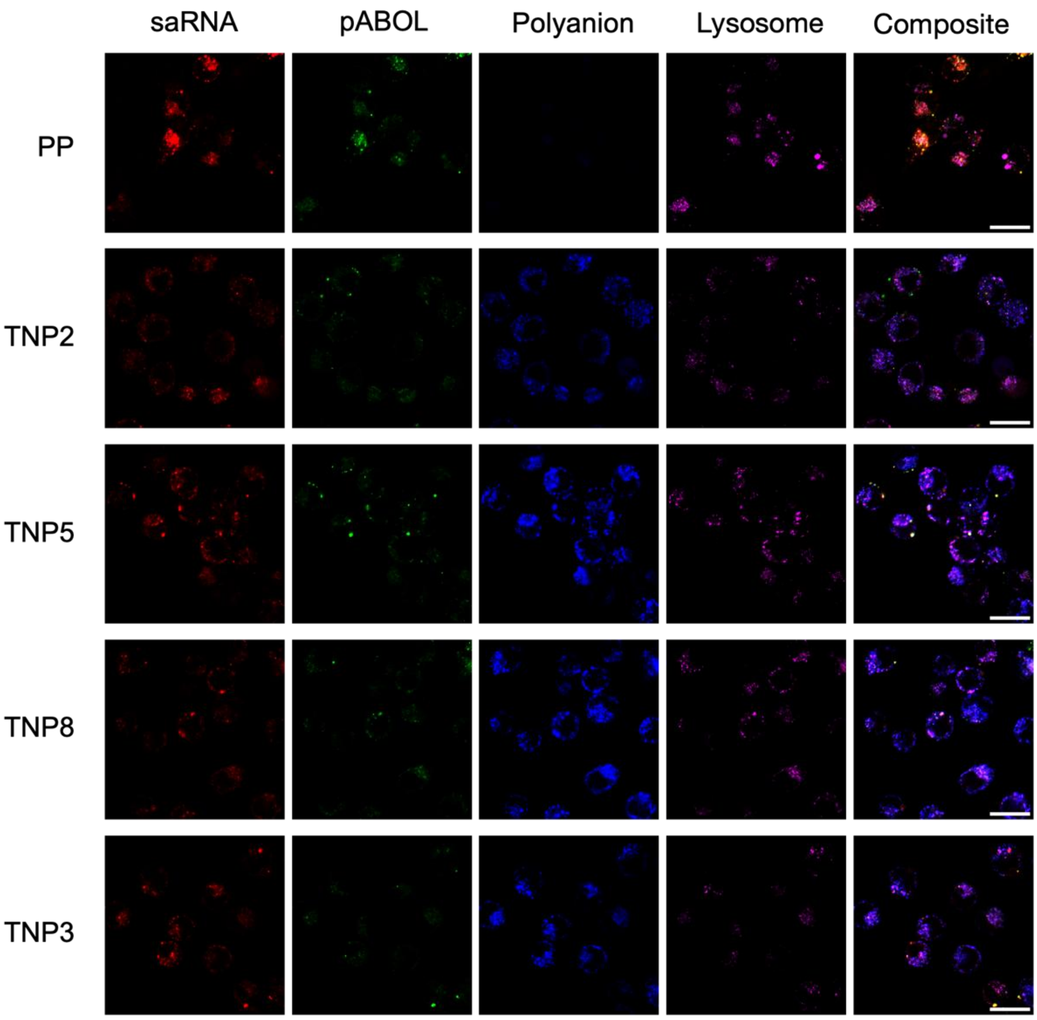

Figure S20. Representative confocal microscopy images of DC2.4 cells treated with PP or TNP containing Cy5-labeled saRNA, FITC-pABOL, and AF405-polyanions for 4 h in the presence of FBS. The acidic compartments were stained with Lysotracker Red. Scale bar = 20 μm.

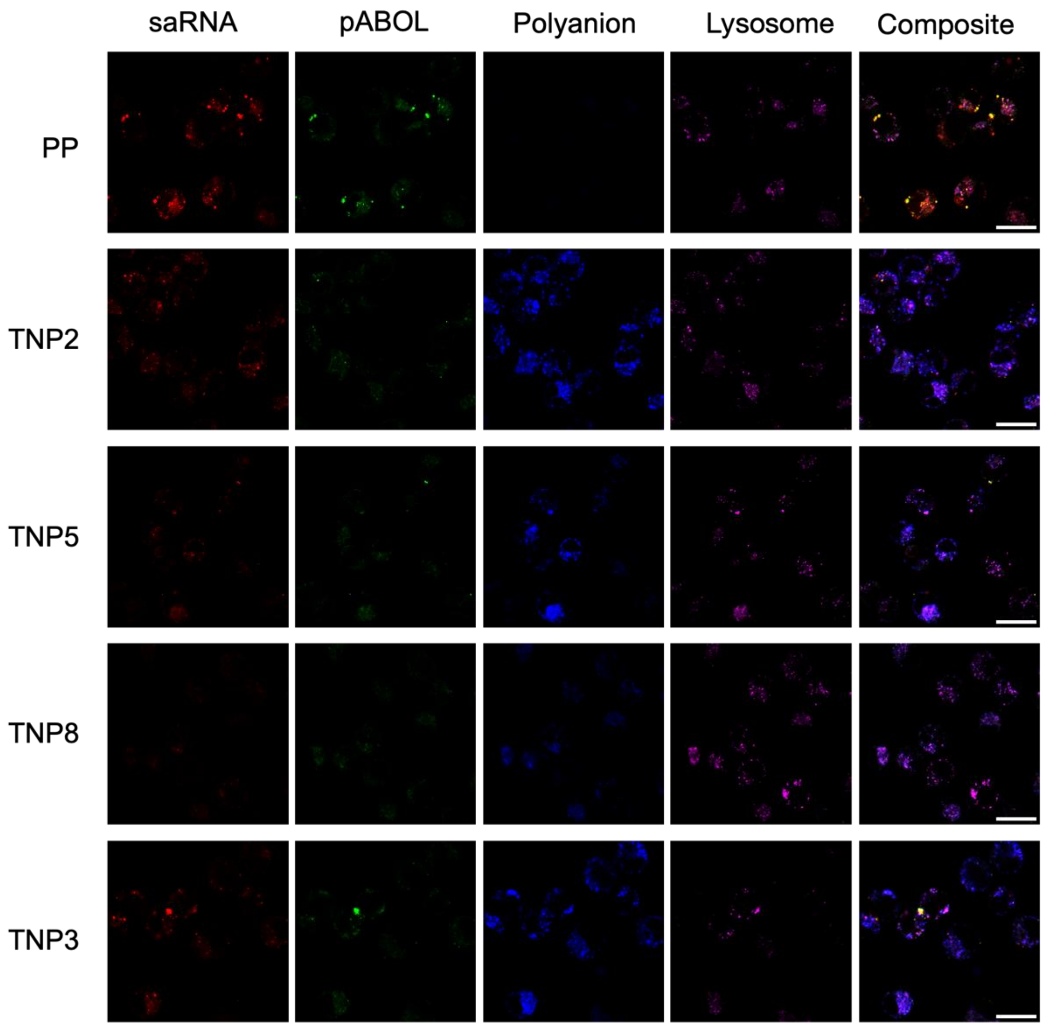

Figure S21. Representative confocal microscopy images of DC2.4 cells treated with PP or TNP containing Cy5-labeled saRNA, FITC-pABOL, and AF405-polyanions for 4 h in the absence of FBS. The acidic compartments were stained with Lysotracker Red. Scale bar = 20 μm.

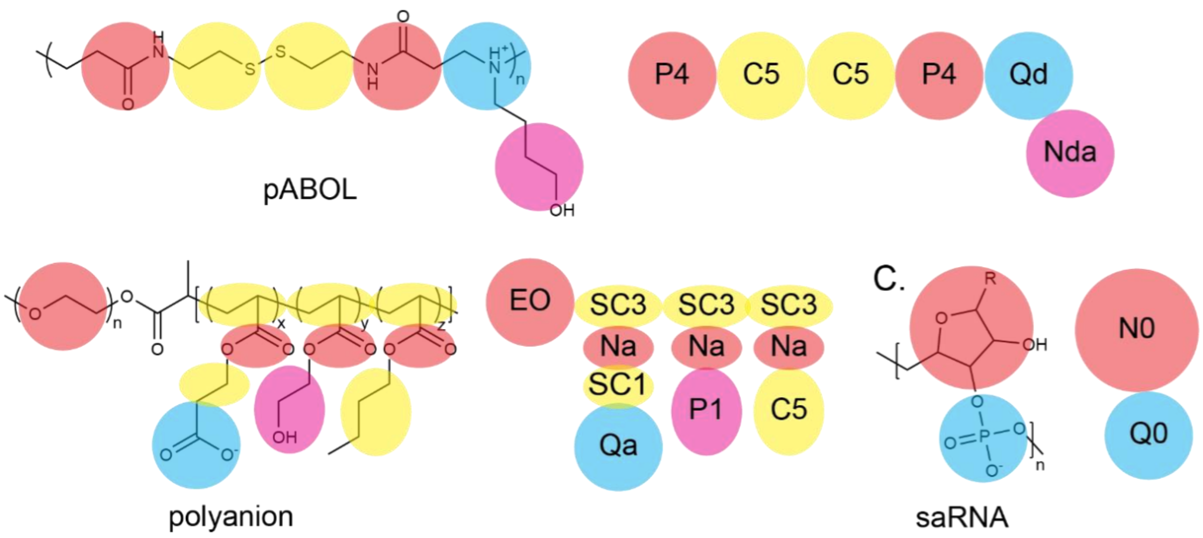

Figure S22. All-atom（AA）and coarse-grained（CG）representations of the polymer structures, with chemical groups color-coded according to their corresponding CG beads. A full description of CG beads used is available in the GROMACS user manual version 5.1.5 (Ref 84).

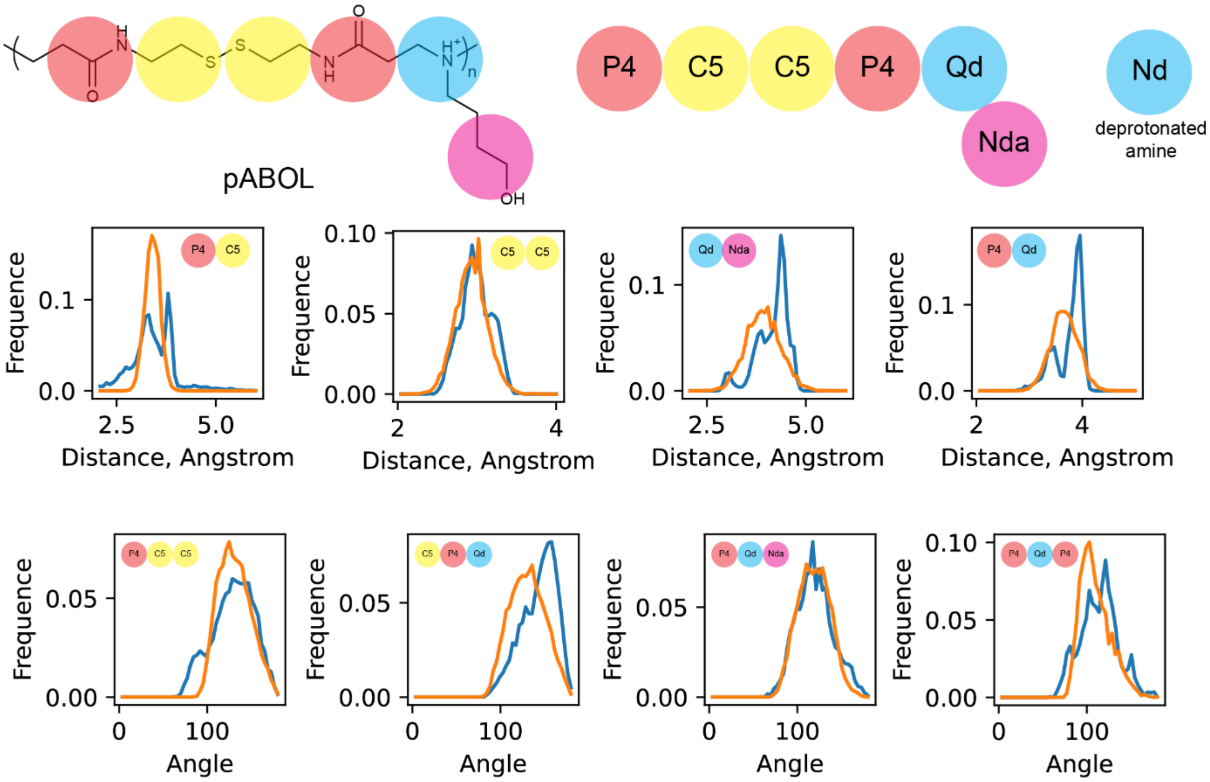

Figure S23. Distributions of bond lengths and angles for pABOL. All plots are labeled with the corresponding beads on the top. Orange lines are from CG simulations, and blue lines are from AA simulations with virtual CG beads.

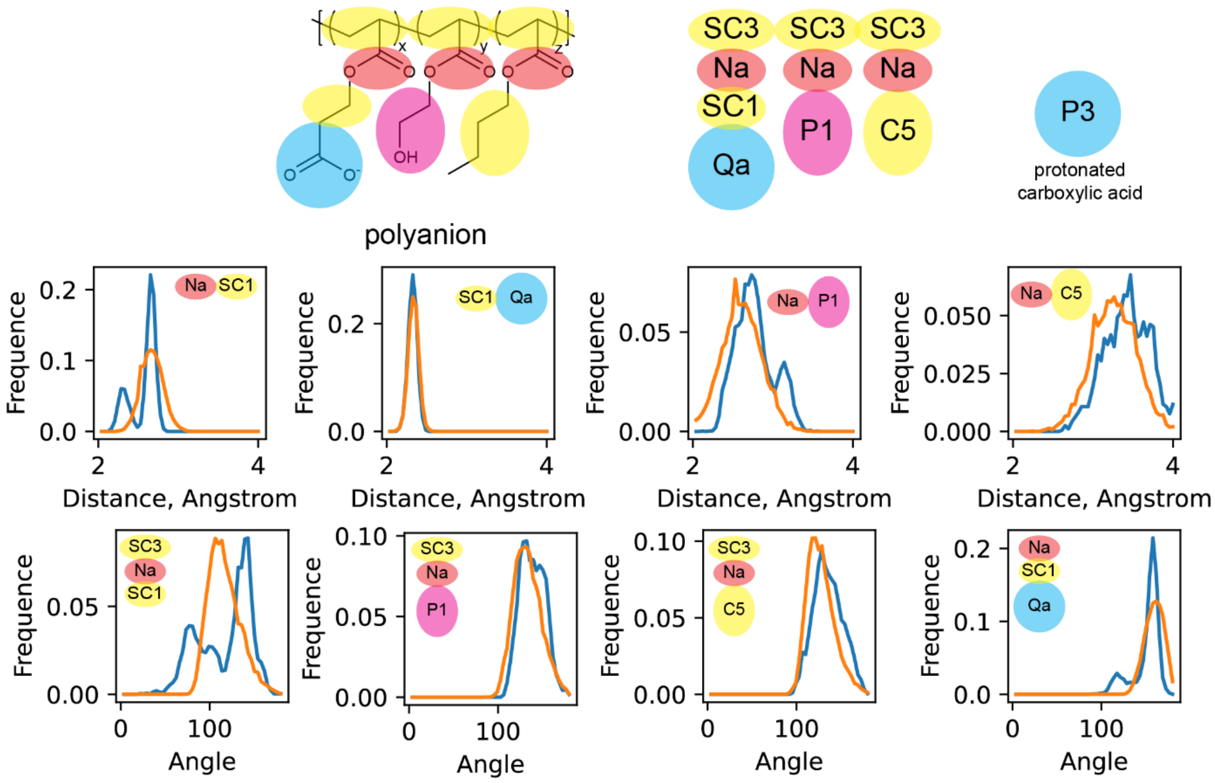

Figure S24. Distributions of bond lengths and angles for polyanions. All plots are labeled with the corresponding beads on the top. Orange lines are from CG simulations, and blue lines are from AA simulations with virtual CG beads.

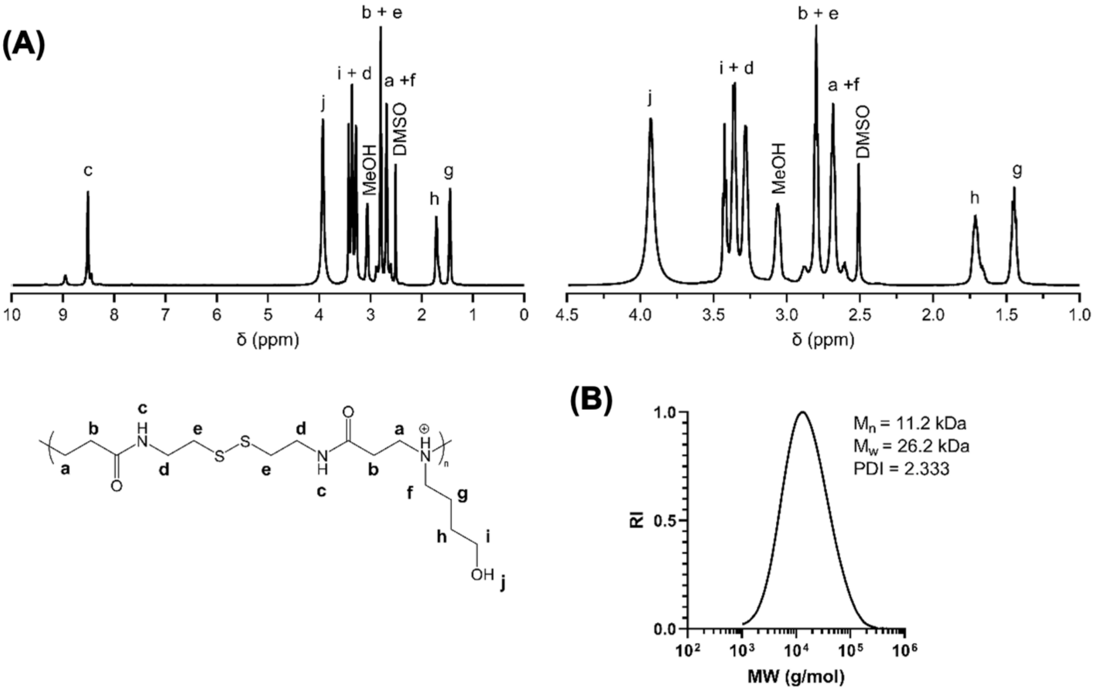

Figure S25. Chemical structure of pABOL, (A) ^{1}H NMR spectra of pABOL in DMSO-*d₆*, and (B) normalized refractive index (RI) trace from GPC of pABOL in LiBr-buffered DMF.

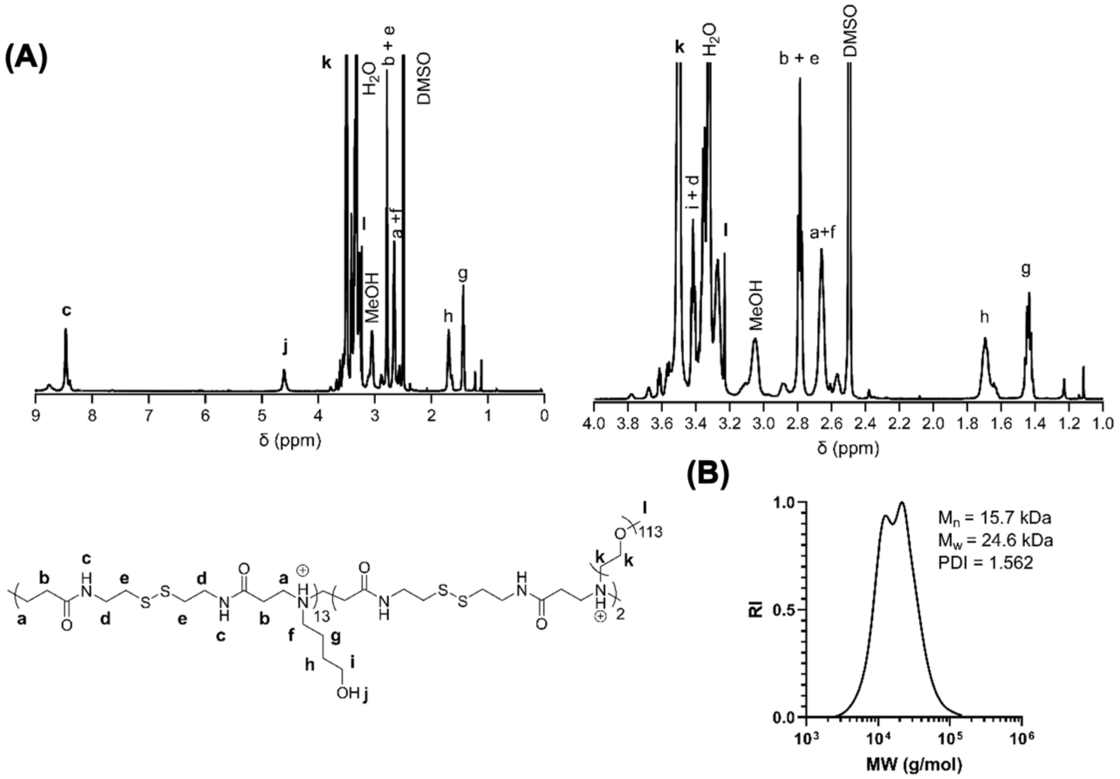

Figure S26. Chemical structure of p(PEG-*co*-ABOL), (A) ^{1}H NMR spectra of p(PEG-*co*-ABOL) in DMSO-*d6*, and (B) normalized refractive index (RI) trace from GPC of p(PEG-*co*-ABOL) in LiBr-buffered DMF.

Table S1. Polyanion library characterization using NMR and GPC.

Polymer	Structure	x	y	z	$M_{n,th}$ (kDa)	$M_{n,GPC}$ (kDa)	Đ
	mPEG$_{45}$-*bl*-p(CEA$_x$-*st*-HEA$_y$)						
P10	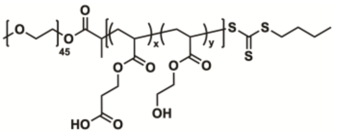	9	27	0	6.7	9.9	1.14
P11		18	18	0	6.9	13.1	1.11
P12		27	9	0	7.2	14.9	1.11
	mPEG$_{45}$-*bl*-p(CEA$_x$-*st*-HEA$_y$-*st*-BA$_z$)						
P13	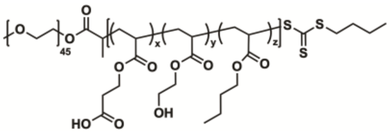	9	14	14	6.9	6.5	1.24
P14		18	9	9	7.0	11.9	1.14
P15		27	4	4	7.1	14.2	1.13
	mPEG$_{45}$-*bl*-p(CEA$_x$-*st*-BA$_z$)						
P16	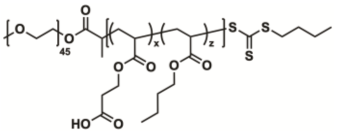	9	0	27	7.0		
P17		18	0	18	7.1	10.4	1.12
P18		27	0	9	7.3	13.8	1.13
	p(OEGA$_9$-*st*-CEA$_x$-*st*-HEA$_y$)						
P19	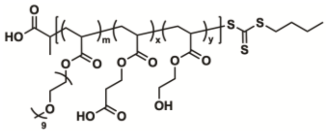	9	27	0	9.0	14.1	1.14
P20		18	18	0	9.2	16.1	1.17
P21		27	9	0	9.5	18.2	1.15
	p(OEGA$_9$-*st*-CEA$_x$-*st*-HEA$_y$-*st*-BA$_z$)						
P22	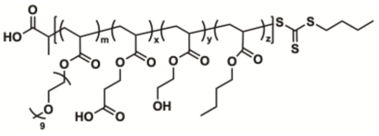	9	14	14	9.3	10.1	1.23
P23		18	9	9	9.4	15.6	1.14
P24		27	4	4	9.4	18.1	1.15
	p(OEGA$_9$-*st*-CEA$_x$-*st*-BA$_z$)						
P25	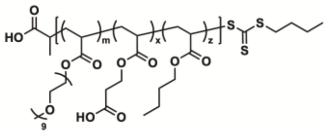	9	0	27	9.3	8.1	1.18
P26		18	0	18	9.5	14.0	1.12
P27		27	0	9	9.6	17.8	1.13
	mPEG$_{113}$-*bl*-p(CEA$_x$-*st*-HEA$_y$)						
P28	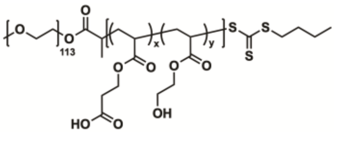	36	36	0	14.6		
P29		54	54	0	19.3		
P30		90	90	0	28.6		
P31		160	160	0	46.9		
	mPEG$_{113}$-*bl*-p(CEA$_x$-*st*-HEA$_y$-*st*-BA$_z$)						
P32	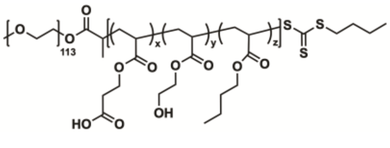	36	18	18	14.8		
P33		54	27	27	19.6		
P34		90	45	45	29.2		
P35		160	80	80	47.8		

Table S2. PP and TNP component neutron scattering length densities (SLD) calculated by the SasView SLD Calculator using their mass densities and chemical formula.

Component	Chemical formula	Density (g/mL)	SLD (×10^{-6} Å^{-2})
pABOL	$(C_{14}H_{25}N_3O_3S_2)_n$	1.00	0.88
p(PEG-*co*-ABOL)	$C_{1110}H_{1269}N_{45}O_{271}S_{30}$	1.02	1.41
P1	$C_{424}O_{232}H_{756}S_3$	1.05	0.88
P2	$C_{442}O_{250}H_{756}S_3$	1.12	1.04
P3	$C_{433}O_{241}H_{756}S_3$	1.14	1.01
P5	$C_{460}O_{232}H_{792}S_3$	1.10	0.95
P8	$C_{487}O_{223}H_{828}S_3$	1.09	0.92
RNA	$C_{89507}H_{101228}N_{36256}O_{65055}P_{9384}$	1.76[1]	3.6[2]/3.45
D_2O	D_2O	1.10	6.34

1. Unruh T, Götz K, Vogel C, et al. Mesoscopic Structure of Lipid Nanoparticle Formulations for mRNA Drug Delivery: Comirnaty and Drug-Free Dispersions. *ACS Nano*. 2024;18:9746-9764.

2. Arteta MY, Kjellman T, Bartesaghi S, et al. Successful reprogramming of cellular protein production through mRNA delivered by functionalized lipid nanoparticles. *Proc Natl Acad Sci U S A*. 2018;115(15):E3351-E3360.

Table S3. Results from the Guinier-Porod / power-law fit of the SANS data for the PP and TNP in different buffer conditions.

Sample	Buffer	Porod_exp	R_g (nm)	s	Fitting q-range	χ^2
PP	pH 6.5 B-T	3.6 ± 0.4	20.8 ± 0.6	0.00 ± 0.04	0.00524-0.0277	0.83
	pH 7.4 PB	4.0 ± 0.3	-	-	0.0043-0.011	0.62
	pH 7.4 PBS	3.3 ± 0.4	-	-	0.00443-0.0106	1.87
TNP1	pH 6.5 B-T	1.9 ± 0.1	-	-	0.00481-0.0181	1.27
TNP3	pH 6.5 B-T	4.5 ± 0.1	8.2 ± 0.1	0.55 ± 0.02	0.00531-0.0457	1.37
TNP2	pH 6.5 B-T	4.5 ± 0.1	6.5 ± 0.1	0.68 ± 0.03	0.00653-0.0564	1.03
	pH 5.5 PB	1.5 ± 0.1	-	-	0.00767-0.0201	1.09
	pH 7.4 PB	4.5 ± 0.1	5.4 ± 0.1	1.33 ± 0.02	0.00638-0.0514	2.04
	pH 7.4 PBS	2.1 ± 0.1	-	-	0.00756-0.0283	1.66
TNP5	pH 6.5 B-T	4.5 ± 0.1	6.8 ± 0.1	0.49 ± 0.02	0.00526-0.0591	1.65
	pH 5.5 PB	1.2 ± 0.0	-	-	0.00562-0.0259	2.07
	pH 7.4 PB	4.5 ± 0.1	6.4 ± 0.1	0.88 ± 0.02	0.00558-0.0484	1.93
	pH 7.4 PBS	1.3 ± 0.0	-	-	0.00448-0.0273	1.49
TNP8	pH 6.5 B-T	4.1 ± 0.2	5.5 ± 0.1	0.64 ± 0.01	0.00557-0.0624	1.31
	pH 5.5 PB	1.6 ± 0.1	-	-	0.00808-0.0263	1.64
	pH 7.4 PB	4.5 ± 0.1	5.8 ± 0.1	0.84 ± 0.02	0.00532-0.0601	1.33
	pH 7.4 PBS	1.3 ± 0.0	-	-	0.00564-0.0269	1.59

Table S4. Results from the core_shell_sphere fit of the SANS data for the PP and TNP in preparation buffer and pH 7.4 PB.

Sample	Buffer	Core		Shell		Fitting q-range	χ^2
		Radius (nm)	SLD (×10^{-6} Å^{-2})	Thickness (nm)	SLD (×10^{-6} Å^{-2})		
PP	pH 6.5 B-T	3.3 ± 0.2	1.23 ± 0.51	17.2 ± 0.3	6.12 ± 0.02	0.00524-0.1	1.38
TNP3	pH 6.5 B-T	9.1 ± 0.0	2.18 ± 0.22	19.1 ± 0.7	6.24 ± 0.00	0.00531-0.1	1.01
TNP2	pH 6.5 B-T	7.4 ± 0.1	1.37 ± 0.22	15.2 ± 0.6	6.20 ± 0.01	0.00653-0.1	2.76
	pH 7.4 PB	8.7 ± 0.1	2.39 ± 0.25	18.6 ± 0.4	6.16 ± 0.01	0.00638-0.1	4.19
TNP5	pH 6.5 B-T	7.4 ± 0.0	1.51 ± 0.21	16.0 ± 0.5	6.24 ± 0.00	0.00526-0.1	2.55
	pH 7.4 PB	8.3 ± 0.0	2.00 ± 0.24	18.4 ± 0.3	6.20 ± 0.01	0.00558-0.1	4.34
TNP8	pH 6.5 B-T	6.3 ± 0.0	1.21 ± 0.19	14.6 ± 0.4	6.22 ± 0.00	0.00557-0.1	10.12
	pH 7.4 PB	7.2 ± 0.0	1.44 ± 0.24	16.8 ± 0.4	6.20 ± 0.06	0.00532-0.1	11.32
PEG-PP	pH 6.5 B-T	3.0 ± 0.1	1.71 ± 0.31	15.0 ± 0.2	6.22 ± 0.00	0.00693-0.1	1.30
	pH 7.4 PB	3.5 ± 0.1	2.53 ± 0.34	16.5 ± 0.1	6.20 ± 0.01	0.00444-0.1	4.31

Table S5. Bead types used in different buffer conditions.

Material	pKa	Charge	pH 5.5		pH 6.5		pH 7.4	
			% Charge	Beads	% Charge	Beads	% Charge	Beads
RNA	2	-	100.0	Q0	100.0	Q0	100.0	Q0
pABOL	7.1	+	97.5	Qd (Nd)	79.9	Qd (Nd)	33.4	Qd (Nd)
P2	5.1	-	69.6	Qa (P3)	95.8	Qa (P3)	99.5	Qa (P3)
P5	5.2	-	68.1	Qa (P3)	95.5	Qa (P3)	99.4	Qa (P3)
P8	5.4	-	56.9	Qa (P3)	92.9	Qa (P3)	99.1	Qa (P3)

Table S6. Number of molecules in simulation system setup.

	PP				TNP2				TNP5				TNP8			
	pH 5.5	pH 6.5	pH 7.4	pH 7.4 PBS	pH 5.5	pH 6.5	pH 7.4	pH 7.4 PBS	pH 5.5	pH 6.5	pH 7.4	pH 7.4 PBS	pH 5.5	pH 6.5	pH 7.4	pH 7.4 PBS
Polyanion	0	0	0	0	35	35	35	35	35	35	35	35	35	35	35	35
pABOL	50	50	50	50	50	50	50	50	50	50	50	50	50	50	50	50
RNA	2	2	2	2	2	2	2	2	2	2	2	2	2	2	2	2
Ion -1	600	550	100	2371	110	150	0	2271	110	150	0	2271	215	150	0	2271
Ion +1	4878	150	4878	2847	4878	415	5478	3447	4878	415	5478	3447	4878	415	5478	3447
Ion -3	1626	0	1626	192	1626	0	1626	192	1626	0	1626	192	1626	0	1626	192
Water	200000	200000	200000	200000	200000	200000	200000	200000	200000	200000	200000	200000	200000	200000	200000	200000
